# Genome-wide deletion mutant analysis reveals genes required for respiratory growth, mitochondrial genome maintenance and mitochondrial protein synthesis in *Saccharomyces cerevisiae*

**DOI:** 10.1186/gb-2009-10-9-r95

**Published:** 2009-09-14

**Authors:** Sandra Merz, Benedikt Westermann

**Affiliations:** 1 Institut für Zellbiologie, Universität Bayreuth, Universitätsstraße 30, 95440 Bayreuth, Germany; 2 Bayreuther Zentrum für Molekulare Biowissenschaften (BZMB), Universität Bayreuth, Universitätsstraße 30, 95440 Bayreuth, Germany

## Abstract

**Background:**

The mitochondrial respiratory chain produces metabolic energy by oxidative phosphorylation. Biogenesis of the respiratory chain requires the coordinated expression of two genomes: the nuclear genome encoding the vast majority of mitochondrial proteins, and the mitochondrial genome encoding a handful of mitochondrial proteins. The understanding of the molecular processes contributing to respiratory chain assembly and maintenance requires the systematic identification and functional analysis of the genes involved.

**Results:**

We pursued a systematic, genome-wide approach to define the sets of genes required for respiratory activity and maintenance and expression of the mitochondrial genome in yeast. By comparative gene deletion analysis we found an unexpected phenotypic plasticity among respiratory-deficient mutants, and we identified ten previously uncharacterized genes essential for respiratory growth (*RRG1 *through *RRG10*). Systematic functional analysis of 319 respiratory-deficient mutants revealed 16 genes essential for maintenance of the mitochondrial genome, 88 genes required for mitochondrial protein translation, and 10 genes required for expression of specific mitochondrial gene products. A group of mutants acquiring irreversible damage compromising respiratory capacity includes strains defective in assembly of the cytochrome *c *oxidase that were found to be particularly sensitive to aging.

**Conclusions:**

These data advance the understanding of the molecular processes contributing to maintenance of the mitochondrial genome, mitochondrial protein translation, and assembly of the respiratory chain. They revealed a number of previously uncharacterized components, and provide a comprehensive picture of the molecular processes required for respiratory activity in a simple eukaryotic cell.

## Abstract 2

A genome-wide deletion mutant analysis in budding yeast reveals genes required for respiratory growth, mitochondrial genome maintenance and mitochondrial protein synthesis.

## Background

Mitochondria are the major sites of metabolic energy production in animals and most other eukaryotic organisms. Electrons generated by the oxidation of nutrients are passed along the respiratory chain and finally transferred to molecular oxygen in a process called oxidative phosphorylation. Energy released by the passage of electrons is stored as a proton gradient across the mitochondrial inner membrane and harvested by the ATP synthase to produce ATP from ADP and phosphate [1]. In an average human individual, ATP is synthesized at an astonishing rate of 9 × 10^20 ^molecules per second, totaling an amount of 65 kg per day [2]. In most eukaryotic organisms, the respiratory chain consists of five multi-subunit complexes: complex I, NADH dehydrogenase; complex II, succinate dehydrogenase; complex III, cytochrome bc_1 _complex; complex IV, cytochrome *c *oxidase; and complex V, ATP synthase [1]. In some organisms, including baker's yeast, *Saccharomyces cerevisiae*, complex I is replaced by an alternative NADH dehydrogenase that consists of a single amino acid chain [3,4].

Biogenesis of the respiratory chain depends on coordinated expression of gene products encoded by the nuclear and mitochondrial genomes. The vast majority of the approximately 1,000 proteins that make up the mitochondrial proteome is encoded by nuclear genes, while a small number of protein-coding genes have been retained in the mitochondrial genome during the evolution of eukaryotic cells - thirteen in humans, eight in *Saccharomyces cerevisiae*, and as little as three in the protist *Plasmodium falciparum *[5]. Proteins encoded by the mitochondrial genome are generally restricted to a few respiratory chain complex subunits and - in some organisms - components required for synthesis and assembly of mitochondria-encoded proteins [5]. In order to express this handful of mitochondrial genes, the cell synthesizes about 200 nuclear-encoded proteins that are devoted to mitochondrial genome maintenance and gene expression [6,7].

*S. cerevisiae *is a powerful model organism to genetically dissect the pathways required for maintenance of respiratory activity because it is capable of satisfying its energy requirements with ATP generated by fermentation. Thus, oxidative phosphorylation and the presence of the mitochondrial genome are dispensable as long as fermentable carbon sources, such as glucose or fructose, are present in the growth medium. Even when oxygen is available, yeast cells generate ATP primarily by glycolysis with ethanol as an end product of fermentation. Most respiratory functions are repressed under these conditions by catabolite repression [8]. Only when fermentable carbon sources become limiting, genes required for respiration are induced, and ATP is generated by metabolizing non-fermentable carbon sources, such as ethanol, glycerol or lactate [9,10]. Yeast mutants defective in oxidative phosphorylation are unable to grow on media containing non-fermentable carbon sources. On media containing limiting amounts of fermentable carbon sources, these mutants form smaller colonies than wild-type strains. The term *petite *has been coined to describe this characteristic phenotype [11]. The originally isolated *petite *mutants that were described in the 1940s were later found to have long deletions in the mitochondrial genome (termed [*rho*^-^]) or completely lack mitochondrial DNA (termed [*rho*^0^]). Mutants with lesions in the mitochondrial genome are referred to as cytoplasmic *petite*, whereas respiratory-deficient strains carrying mutations in the nuclear genome are referred to as nuclear *petite *or *pet *mutants [12]. Nuclear *pet *genes include, but are not limited to, genes encoding respiratory chain components, factors required for folding and assembly of respiratory chain subunits, proteins required for mitochondrial DNA (mtDNA) inheritance, mitochondrial RNA and protein synthesis, and components of the machinery determining mitochondrial morphology [12-14].

By the end of the last century, more than 200 complementation groups and more than 100 *pet *genes had been identified by classic yeast genetic methods [12,13,15]. The availability of the yeast gene deletion library nowadays allows systematic and comprehensive screens to assign functions to almost all of the approximately 4,800 non-essential yeast genes [16]. Here, we aimed at a large-scale functional analysis of respiratory-deficient yeast mutants to define the complement of genes a yeast cell requires for mitochondrial gene expression and respiratory activity. Comparative gene deletion analysis revealed a surprising phenotypic plasticity of respiratory-deficient mutants and allowed us to identify ten novel genes that are essential for respiratory activity in yeast. By systematic functional tests of respiratory-deficient mutants we obtained a comprehensive picture of the molecular processes required for respiratory activity and maintenance and expression of the mitochondrial genome in yeast.

## Results and discussion

### Genes required for respiratory growth

**Figure 1:**
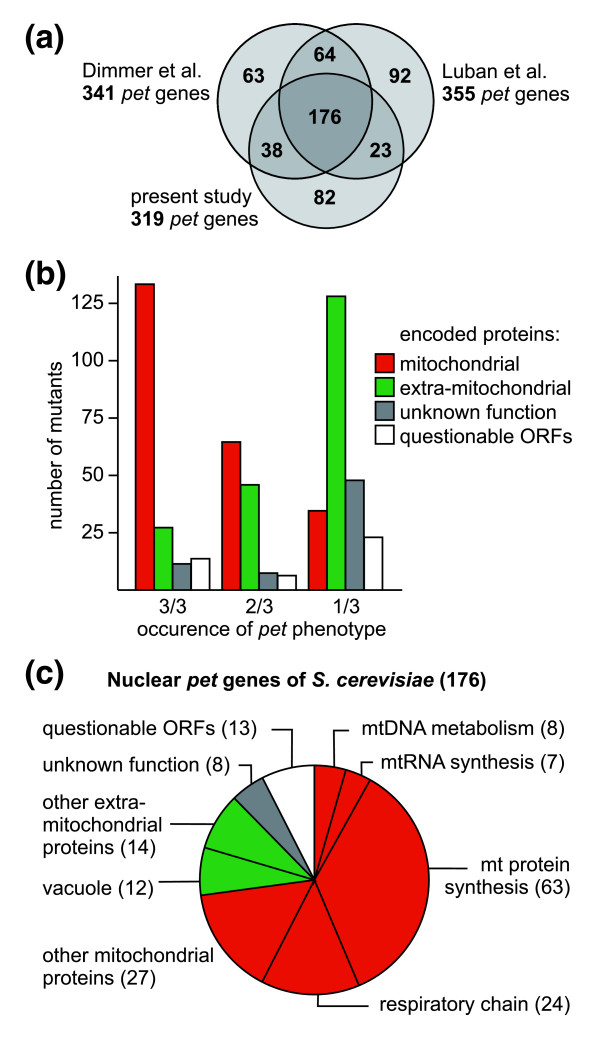
Nuclear *pet *genes of *S. cerevisiae*. **(a) **The numbers of *pet *mutants identified in three screens of the yeast deletion library are indicated. References: Dimmer *et al*. [14], Luban *et al*. [17]. **(b) **The intracellular location of proteins encoded by *pet *genes has been grouped according to their frequency of occurrence in screens of the deletion library. The graph is a summary of data contained in Additional data file 3. **(c) **Cellular functions of proteins encoded by highly penetrant *pet *genes. Functions have been assigned according to data from the Saccharomyces Genome Database [19] and manual annotation. Red indicates mitochondrial proteins, green known extra-mitochondrial proteins, grey unknown proteins, and white dubious ORFs overlapping with known protein-coding genes.

Two independent screens of the yeast deletion library have previously revealed two partially overlapping sets of *pet *genes. By plating the homozygous diploid yeast deletion library on media containing glycerol as a carbon source, Dimmer *et al*. [14] identified 341 deletion mutants that were unable to grow. In a very similar approach, Luban *et al*. [17] identified a set of 355 respiratory-deficient clones by screening the *MAT*a yeast deletion library. While about two-thirds of the mutants in each screen were found to be respiratory-deficient also in the other screen, a surprisingly large number of mutants were isolated only once [17]. It seems unlikely that this is due to differences in the genetic background, because both screens have been conducted in largely isogenic strains, BY4743 and BY4741 [18]. Here, we screened the *MAT*α deletion library (BY4742 background) to obtain a third set of respiratory-deficient mutants. This was then compared with the data obtained by Dimmer *et al*. [14] and Luban *et al*. [17]. The *MAT*α deletion library contained 319 mutants that were unable to grow on glycerol-containing medium (Additional data file 1). Of these, 176 are common to all three sets of *pet *genes (Figure 1a). In the following we will refer to these genes as highly penetrant *pet *genes. 125 genes have been identified in two of three screens, and 237 genes have been identified only once (*pet *genes unique to this study are listed in Additional data file 2). Nineteen additional *pet *genes (not included in the set of 176 highly penetrant *pet *genes) were only covered by one or two libraries. Based on data from the Saccharomyces Genome Database [19] and manual annotation, we grouped all genes according to their frequency of occurrence in *pet *screens and the intracellular location and function of their gene products (Additional data file 3).

Strikingly, 129 out of the 176 *pet *genes found in all three screens encode proteins known to be located in mitochondria, corresponding to 73.3% (Figure 1b; Additional data file 3). The fraction of genes encoding mitochondrial proteins was reduced to 52.1% for *pet *genes found in two of three screens, and as low as 14.7% for *pet *genes that were found only once (Figure 1b; Additional data file 3). This demonstrates a clear correlation of the penetrance of *pet *phenotypes with mitochondrial functions of the affected gene products. The majority of the 176 *pet *genes found in all libraries encode proteins devoted to maintenance and expression of the mitochondrial genome and assembly of the respiratory chain (Figure 1c; Additional data file 3). Thirteen open reading frames (ORFs) are unlikely to encode proteins, because they overlap with other known genes (Additional data file 3), reducing the number of protein-coding genes to 163.

Differences of growth behavior of strains taken from different versions of the deletion libraries could either reflect inherent properties of the mutant strains, they could be due to technical differences between the various screens, or they could mean that a given deletion in one collection is wrong (as it has occasionally been observed by us and others; for example, strains not correct in the *MAT*α library include Δ*rpo41 *lacking the mitochondrial RNA polymerase). We reasoned that incorrect mutants will be enriched among strains that showed respiratory competence in one screen but were respiratory-deficient in the two other screens because it is more likely that a specific phenotype is obscured rather than generated by chance. To test this, we checked the genotypes of 29 mutants taken from the *MAT*α library by PCR. Nineteen randomly chosen mutants were tested that were respiratory-competent in the *MAT*α library, but respiratory-deficient in the *MAT*a and homozygous diploid library. Of these, six mutants (Δ*yal012w*, Δ*ybl038w*, Δ*ydl202w*, Δ*ydr268w*, Δ*yor205c*, and Δ*ypl029w*) contained exclusively the wild-type allele, seven mutants (Δ*ydr231c*, Δ*ydr332w*, Δ*yil036w*, Δ*yjr090c*, Δ*ymr066w*, Δ*ypr047w*, and Δ*ypr124w*) contained a mixture of deletion and wild-type alleles, and six mutants were found to have the correct genotype (Δ*yal047c*, Δ*ybr163w*, Δ*ydr323c*, Δ*ykl148c*, Δ*yml081c-a*, and Δ*yml129c*). In addition, we tested ten mutants that showed a *pet *phenotype only in the *MAT*α library, but not in the *MAT*a and homozygous diploid library, and ten mutants, that showed a *pet *phenotype in all three screens. All mutants of the two latter groups were found to have the correct genotype. This means whenever a wrong deletion was detected, a *pet *phenotype was obscured by the presence of the wild-type allele, whereas all respiratory-deficient mutants tested were found to have the correct genotype. We conclude that several discrepancies of growth phenotypes can be ascribed to wrong genotypes that are present in the deletion library. However, the fact that a relatively large number of mutants with confirmed correct genotypes show differences in their growth behavior points to a pronounced phenotypic plasticity of *pet *mutants. Furthermore, the correlation of the penetrance of *pet *phenotypes with mitochondrial localization of gene products (Figure 1b) is a clear indication that the phenotypic variability is not only due to wrong deletions present in the mutant libraries, but also reflects biological processes.

Eight highly penetrant *pet *genes (*YDR065w*, *YGR150c*, *YJL046w*, *YLL033w*, *YLR091w*, *YMR293c*, *YOR305w*, and *YPR116w*) encode previously uncharacterized proteins, and earlier studies have revealed a respiratory-deficient phenotype for two additional ORFs of unknown function that were not covered by all three yeast deletion libraries, *YNL213c *[20] and *YJL062w-a *[21]. We confirmed the identity of these mutant strains by PCR and named the genes *RRG1 *through *RRG10 *(for 'Required for respiratory growth') as the functional analysis described below proves that their products are novel factors required for respiratory growth.

### Comparative growth analysis on different non-fermentable carbon sources

We asked whether the respiratory-deficient phenotype observed for the 319 *pet *mutants isolated from the *MAT*α deletion library is specific to glycerol metabolism or reflects a general lack of respiration competence. To test this, we plated the mutants also on complete media containing lactate or ethanol as sole carbon sources. The vast majority (305 strains, corresponding to 95.6% of the *pet *mutants) failed to grow on all non-fermentable carbon sources that were tested. Of the remainder, seven mutants showed a growth defect only on glycerol-containing medium, seven on glycerol or ethanol-containing media, and one mutant on glycerol or lactate containing media (Additional data file 4). As *pet *phenotypes are highly reproducible even on different carbon sources we conclude that our screen gives a largely accurate estimate of respiratory deficiencies in the *MAT*α deletion library.

### Restoration of respiratory activity by mating with Δ*mip1 *and by cytoduction of [rho^+^] mitochondria

**Figure 2:**
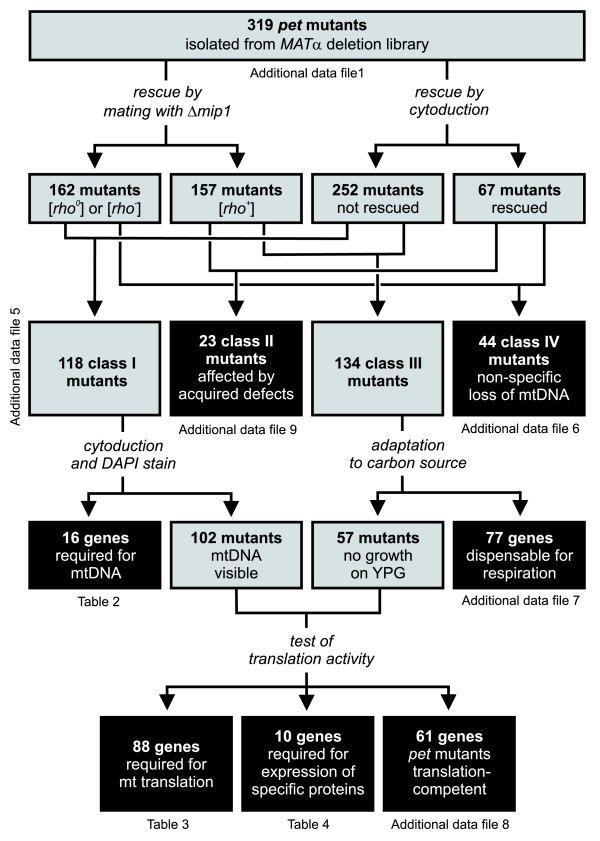
Summary of the systematic functional analysis of 319 *pet *mutants isolated from the *MAT*α yeast deletion library. Grey boxes indicate groups of mutants that were further analyzed, black boxes indicate the final level of resolution of functional analysis. See text for details.

In order to define the genetic basis of respiratory deficiency, we subjected the complete set of 319 *pet *mutants isolated from the *MAT*α deletion library to various functional tests (Figure 2). As a *petite *phenotype is often associated with the complete or partial loss of the mitochondrial genome [13], we first asked whether the *pet *mutants contain functional mtDNA. To test this, *pet *mutants were mated with a strain lacking the mtDNA polymerase Mip1. As the Δ*mip1 *strain is [*rho*^0^] [22], resulting heterozygous diploid strains are able to grow on glycerol-containing medium only if functional mtDNA is provided by the *pet *mutant mating partner. We observed restoration of respiratory activity in 157 heterozygous diploid strains demonstrating that the parental *pet *strains possessed an intact mitochondrial genome. In contrast, 162 strains failed to grow on glycerol-containing medium after mating, suggesting that the parental *pet *mutants were [*rho*^-^] or [*rho*^0^].

The complementation test with Δ*mip1 *does not discern whether the protein encoded by the *pet *gene is obligatorily required for maintenance of mtDNA, or whether a functional mitochondrial genome had been spontaneously lost in the *pet *mutant during many generations of growth. To discriminate between these possibilities, we replenished cells with mitochondria containing a wild-type [*rho*^+^] mitochondrial genome by cytoduction. In brief, *pet *mutants were crossed with a [*rho*^+^] donor strain that carries a *kar1 *mutation to prevent karyogamy in the zygote. After counterselection against nuclear chromosomes of the donor strain, growth of the haploid progeny was assessed on glycerol-containing media. Restoration of respiratory activity after cytoduction was observed in 67 *pet *mutants, whereas 252 strains failed to grow on non-fermentable carbon sources.

**Figure 3:**
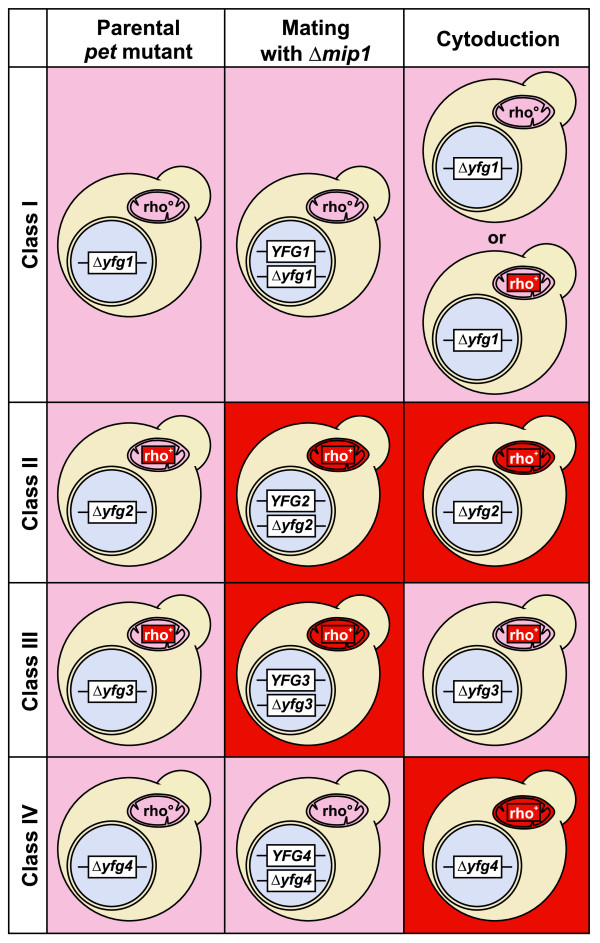
Classes of *pet *mutants. The left column indicates genotypes of haploid *pet *mutant strains taken from the deletion library carrying a deletion in the nuclear genome (Δ*yfg1*, 'your favourite gene 1') and either no mtDNA ([*rho*^0^]; alternatively these strains might be [*rho*^-^]) or a wild type-like mitochondrial genome ([*rho*^+^]; labeled in red). The middle column indicates genotypes of heterozygous diploid strains after mating with Δ*mip1*. The right column indicates genotypes of haploid deletion mutants after having received [*rho*^+^] mitochondria from a donor strain by cytoduction. Respiratory-competent mitochondria are labeled in red, and respiratory-competent yeast cells are depicted on a red background. Class I mutants contain either [*rho*^+^] or [*rho*^0^] or [*rho*^-^] mitochondria after cytoduction. See text for details.

Combining the results from the Δ*mip1 *mating test and the cytoduction experiment allowed us to define four classes of *pet *mutants (Figure 3; Additional data file 5). Class I mutants were not rescued either by mating with Δ*mip1 *or by cytoduction; class II mutants were rescued by mating with Δ*mip1 *as well as by cytoduction; class III mutants were rescued only by mating with Δ*mip1*, but not by cytoduction; and class IV mutants were rescued only by cytoduction, but not by mating with Δ*mip1*. The basic properties of these classes are summarized in Table 1. In the following, the various classes of *pet *mutants are further examined (Figure 2).

**Table 1:** Classes of *pet *mutants

**Class**	**Respiration after mating with Δ*mip1***	**Respiration after cytoduction**	**Associated gene functions**
I	-	-	Genes essential for maintenance of mtDNA (16 mutants); or genes essential for respiration with gradual loss of mtDNA (102 mutants)
II	+	+	Additional effects of extra-genomic factors and/or acquired mitochondrial damage (23 mutants)
III	+	-	Genes essential for respiration but not for maintenance of mtDNA (134 mutants)
IV	-	+	Genes dispensable for respiration, gradual loss of mtDNA (44 mutants)

### Genes required for maintenance of mtDNA

The 118 class I mutants were [*rho*^-^] or [*rho*^0^] and remained respiratory-deficient after introduction of functional mitochondria. This group of mutants is expected to include all components that are essential for maintenance of a [*rho*^+^] genome. In addition, we expected it to contain components deletion of which leads to a gradual loss of mtDNA and, at the same time, induces respiratory deficiency due to lack of functions not directly related to mtDNA maintenance. To discern between these possibilities, we subjected all class I mutants to various functional tests. First, we tested for the presence of mtDNA by DAPI (4',6-diamidino-2-phenylindole) staining immediately after cytoduction. Second, we tested growth on YPG medium after adaptation to the medium by pre-culture on YPG containing low amounts of glucose. And third, we tested mitochondrial protein translation activity by SDS-PAGE and autoradiography after labeling cycloheximide-treated cells with ^35^S methionine.

Genes essential for maintenance of mtDNA were defined by the following criteria: At least 95% of the cells observed by DAPI staining after cytoduction were devoid of mtDNA and the remainder contained less than five mtDNA nucleoids per cell. This phenotype was observed after cytoduction in the Δ*mip1 *mutant lacking the mtDNA polymerase and, therefore, is indicative of instantaneous loss of mtDNA. In addition, cells lacking genes essential for maintenance of mtDNA were expected to be unable to grow on YPG after adaptation to the carbon source, and they were unable to produce even trace amounts of mitochondria-encoded proteins. Sixteen mutants were identified that matched these criteria (Table 2). We propose that the gene products lacking in these mutants are particularly important for maintenance of mtDNA. As expected, this group includes several components known to be involved in mtDNA metabolism: the mtDNA polymerase Mip1 [22]; mtDNA helicases Hmi1 [23] and Pif1 [24]; Apn1, a DNA repair protein active in the nucleus and mitochondria [25]; and aconitase, Aco1, an enzyme of the citric acid cycle that has an additional role in mtDNA maintenance [26].

**Table 2:** Genes essential for maintenance of mtDNA

**Genes encoding components involved in mtDNA metabolism**		
*YKL114c	*APN1*	Involved in repair of DNA damage; located in nucleus and mitochondria
*YLR304c*	*ACO1*	Aconitase; also independently required for mtDNA maintenance
*YML061c*	*PIF1*	DNA helicase; active in nucleus and mitochondria
*YOL095c*	*HMI1*	Mitochondrial inner membrane localized DNA helicase
*YOR330c*	*MIP1*	Catalytic subunit of the mitochondrial DNA polymerase
		
**Genes encoding components involved in mitochondrial transcription and translation**		
*YBR268w	*MRPL37*	Mitochondrial ribosomal protein
*YCR024c	*SLM5*	Mitochondrial asparaginyl-tRNA synthetase
*YDR175c	*RSM24*	Mitochondrial ribosomal protein of the small subunit
*YHR168w*	*MTG2*	Associates with mitochondrial ribosome; possible role in ribosome assembly
*YKL169c		Dubious ORF; partially overlaps with *MRPL38*
*YMR228w*	*MTF1*	Mitochondrial RNA polymerase specificity factor
		
**Genes encoding components involved in oxidative phosphorylation**		
*YDR079w	*PET100*	Specifically facilitates the assembly of cytochrome *c *oxidase
*YPL078c*	*ATP4*	Subunit b of the stator stalk of mitochondrial F_1_F_0 _ATP synthase
		
**Other genes**		
*YGL240w	*DOC1*	Required for the ubiquitination activity of the anaphase promoting complex
*YLR091w	*RRG5*	Unknown function
*YOR211c*	*MGM1*	Mitochondrial GTPase involved in fusion

The list indicates systematic and standard names of genes essential for maintenance of newly re-introduced mtDNA in class I *pet *mutants. The cellular roles of the proteins are indicated according to the Saccharomyces Genome Database [19] or manually annotated. Genes that were previously not known to be essential for maintenance of mtDNA are indicated with an asterisk.

It has been observed that a block of mitochondrial protein synthesis leads to a rapid and quantitative loss of mtDNA [27]. However, the reasons for this phenomenon are still unknown. Here, we observed instantaneous loss of mtDNA in cells lacking Mrpl37, Mtf1, Mtg2, Rsm24, and Slm5, which are all required for mitochondrial transcription or translation, and in a deletion mutant lacking the dubious ORF *YKL091w*, which overlaps with the *MRPL38 *gene encoding a mitochondrial ribosomal protein. Loss of mtDNA at a relatively high rate was also observed in several other class I mutants lacking components of the mitochondrial protein synthesis machinery. These findings underscore the importance of mitochondrial protein synthesis for maintenance of mtDNA. Moreover, rapid loss of mtDNA was observed in the Δ*atp4 *mutant lacking ATPase subunit b. This is consistent with earlier observations [28]; however, the molecular reasons are not understood [29]. Also, Δ*pet100 *mutants lacking a factor required for cytochrome *c *oxidase assembly showed rapid loss of mtDNA. As loss of mtDNA in Δ*atp4*, Δ*mrpl37*, Δ*mtf1*, Δ*mtg2*, Δ*pet100*, Δ*rsm24*, and Δ*slm5 *occurs instantaneously (as rapid as in Δ*mip1*) we consider it likely that replication and/or inheritance of mtDNA is actively suppressed in these strains. These results point to an active role of Atp4, Mrpl37, Mtf1, Mtg2, Pet100, Rsm24, and Slm5 in regulating mtDNA abundance in yeast mitochondria.

Other factors required for mtDNA inheritance are Mgm1, Doc1 and the newly identified protein Rrg5. Mgm1 is a dynamin-related protein required for mitochondrial genome maintenance by mediating mitochondrial fusion [30-32]. Doc1 is involved in cyclin proteolysis as a processivity factor required for the ubiquitination activity of the anaphase promoting complex (APC) [33]. Intriguingly, Doc1 has been found in the mitochondrial proteome [6,34]. Thus, it is tempting to speculate that it links mtDNA replication and/or inheritance to the cell cycle. The *RRG5 *gene (*YLR091w*) encodes a protein of unknown function. Its sequence does not show similarities to any characterized protein. As the Rrg5 protein has been localized to mitochondria [6,34,35], we propose that it is a novel factor essential for maintenance of mtDNA.

In addition to class I mutants, 44 *pet *mutants were identified that were not complemented by mating with Δ*mip1 *but could be rescued by cytoduction. These strains are able to maintain newly re-introduced mtDNA when they are grown on non-fermentable carbon sources (class IV; Additional data file 5). It is conceivable that these mutants have a tendency to spontaneously lose their mitochondrial genome when they are grown on fermentable carbon sources for longer times. This has been observed previously for Δ*mdm31 *and Δ*mdm32 *mutants that showed a *pet *phenotype in the screen performed by Dimmer *et al*. [14], but not in the screens performed by Luban *et al*. [17] and in the screen reported here. Freshly made Δ*mdm31 *and Δ*mdm32 *deletion mutants have been found to be able to maintain [*rho*^+^] mtDNA [36]. However, mtDNA is not stably inherited and is gradually lost after several generations of growth in glucose-containing medium [36]. To test this systematically for all class IV mutants, we replenished mtDNA by cytoduction and then passaged the strains in liquid YPD medium for 10 days to allow for loss of mtDNA. Presence or absence of mtDNA was assayed by DAPI staining immediately after cytoduction and after 10 days of replicative growth. In all strains, at least 90% of the cells contained mtDNA directly after cytoduction. Continued growth in glucose-containing medium led to increased loss of mtDNA in many mutants (Additional data file 6), suggesting that gradual loss of mtDNA accounts for the *pet *phenotype in many class IV mutants. Only few mutants maintained mtDNA as stably as the wild type (Additional data file 6). We consider it possible that these mutants require more generation times or special growth conditions to induce loss of mtDNA, or that these mutants rapidly accumulate mtDNA point mutations or deletions rendering the mitochondrial genome non-functional over time. Interestingly, 77% of the class IV mutants have not been found in the screens by Dimmer *et al*. [14] and Luban *et al*. [17], suggesting that many of the affected genes are only indirectly related to maintenance of respiratory activity.

### Genes required for protein translation in mitochondria

Next, we asked which genes are required for mitochondrial protein synthesis. Mutants defective in this process are expected to be found in either class I or class III. Class I contains mutants that have lost their mtDNA as a consequence of blocked mitochondrial translation activity, whereas class III contains mutants that are defective in translation but maintain an intact mitochondrial genome. In order to be able to test for mitochondrial protein synthesis activity, we replenished wild-type mtDNA in class I mutants by cytoduction. After this treatment, mtDNA could be visualized by DAPI staining in 102 mutants, whereas 16 mutants lacking genes essential for maintenance of mtDNA immediately became [*rho*^0^] (see above; Table 2). For class III mutants, we reasoned that some strains might be unable to grow on medium containing glycerol as the sole carbon source because of synergistic effects of compromised mitochondrial function in combination with catabolite repression, which reduces the expression of genes required for respiration [8]. Therefore, we first relieved catabolite repression in all class III mutants by growth on glycerol-containing medium supplemented with limiting amounts of fermentable carbon source (3% glycerol/0.1% glucose) before replicating the strains on glycerol-containing medium. After this treatment, 77 strains were able to grow on plates containing glycerol as the sole carbon source (Additional data file 7). We conclude that the gene products lacking in these mutants are dispensable for respiration.

Then, we tested mitochondrial translation in a total number of 159 deletion mutants (102 class I mutants with replenished mtDNA and 57 class III mutants unable to grow on glycerol-containing medium after adaptation to the carbon source). Strains were grown to logarithmic growth phase in medium containing fermentable carbon sources, before cytosolic translation was stopped by the addition of cycloheximide. Newly synthesized mitochondrial proteins were labeled with ^35^S methionine, and cell extracts were analyzed by SDS-PAGE and autoradiography.

Mitochondrial translation products could not be detected in 88 mutants (Table 3). We conclude that these genes are required for mitochondrial protein synthesis. Encoded proteins include 39 subunits of the mitochondrial ribosome and several additional components required for mitochondrial transcription, translation or assembly of the respiratory chain [37]. In addition, mitochondrial translation activity was absent in several mutants lacking proteins known to be required for mtDNA inheritance, such as Fzo1 [38,39], Mhr1 [40], Msh1 [41], or Mgm101 [42]. Supposedly, in these strains - and likely also in other class I mutants - the mitochondrial genome had been largely lost or damaged during growth of the strains in the time between cytoduction and the labeling reaction. It should be noted that strain-dependent effects might also play a role, because, for example, Δ*pet309 *was observed to be completely translation-inactive here, whereas mitochondrial translation products could be observed when this mutant was constructed in the W303 genetic background [43]. Five genes (*RRG1*, *YGR102c*, *RRG2*, *RRG6*, and *RRG8*) encode uncharacterized proteins, and two dubious ORFs (*YDR114c *and *YNL184c*) overlap with genes encoding mitochondrial ribosomal proteins. A possible role of Rrg1, Rrg2, Rrg6, and Rrg8 as novel components required for mitochondrial protein synthesis is discussed below.

**Table 3:** Genes essential for mitochondrial translation

**Genes encoding mitochondrial ribosomal proteins**		*YBL090W/MRP21; YBR146W/MRPS9; YBR251W/MRPS5; YBR282W/MRPL27; YCR003W/MRPL32; YCR071C/IMG2; YDL045W-A/MRP10; YDR115W; YDR337W/MRPS28; YDR347W/MRP1; YEL050C/RML2; YER050C/RSM18; YGL129C/RSM23; YGR076C/MRPL25; YGR215W/RSM27; YGR220C/MRPL9; YHR147C/MRPL6; YJL063C/MRPL8; YJL096W/MRPL49; YKL003C/MRP17; YKL138C/MRPL31; YKL155C/RSM22; YKL170W/MRPL38; YKR006C/MRPL13; YKR085C/MRPL20; YLR312W-A/MRPL15; YLR439W/MRPL4; YMR158W/MRPS8; YMR188C/MRPS17; YMR193W/MRPL24; YMR286W/MRPL33; YNL081C/SWS2; YNL177C/MRPL22; YNL185C/MRPL19; YNL252C/MRPL17; YNR037C/RSM19; YOR150W/MRPL23; YOR158W/PET123; YPL173W/MRPL40*
		
**Other genes encoding known proteins**		
*YBL019w	*APN2*	Class II abasic (AP) endonuclease involved in repair of DNA damage
*YBR179c	*FZO1*	Transmembrane GTPase required for mitochondrial fusion
*YDL044c*	*MTF2*	Mitochondrial protein involved in mRNA splicing and protein synthesis
*YDR194c	*MSS116*	Mitochondrial RNA helicase, required for splicing of group II introns
*YDR296w	*MHR1*	Involved in repair, recombination and maintenance of mitochondrial DNA
*YER145c	*FTR1*	Iron permease that mediates high-affinity iron uptake
*YER154w	*OXA1*	Component of the mitochondrial protein export machinery
*YGL071w	*RCS1*	Transcription factor regulates genes involved in iron uptake and cell size
*YGL143c*	*MRF1*	Mitochondrial peptide chain release factor
*YGR171c*	*MSM1*	Met-tRNA synthetase, mitochondrial
*YHL038c*	*CBP2*	Mitochondrial splicing factor
*YHR011w*	*DIA4*	tRNA synthetase, may be involved in mitochondrial function
*YHR038w*	*RRF1*	Mitochondrial ribosome recycling factor
*YHR051w	*COX6*	Cytochrome *c *oxidase subunit VI
*YHR091c*	*MSR1*	Arginyl-tRNA synthetase of mitochondria
*YHR120w	*MSH1*	Involved in mitochondrial DNA repair
*YJL102w*	*MEF2*	Mitochondrial translation elongation factor
*YJL209w	*CBP1*	Required for *COB *mRNA stability or 5' processing
*YJR144w	*MGM101*	Mitochondrial genome maintenance protein
*YKL016c	*ATP7*	ATP synthase subunit d
*YKL134c	*OCT1*	Mitochondrial intermediate peptidase
*YKL194c*	*MST1*	Mitochondrial threonyl tRNA synthase
*YLR067c*	*PET309*	Specific translational activator for the *COX1 *mRNA
*YLR069c*	*MEF1*	Mitochondrial translation elongation factor G
*YLR070c	*XYL2*	Xylitol dehydrogenase
*YLR139c*	*SLS1*	Protein involved in mitochondrial metabolism
*YLR295c	*ATP14*	ATP synthase subunit h
*YMR064w*	*AEP1*	Required for accumulation of transcript of *ATP9/OLI1*
*YMR089c	*YTA12*	Involved in proteolytic and chaperone activities in the inner membrane
*YMR097c*	*MTG1*	Likely functions in assembly of the large ribosomal subunit
*YMR098c	*ATP25*	Required for the stability of *ATP9 *mRNA
*YMR267w	*PPA2*	Inorganic pyrophosphatase, mitochondrial
*YMR287c	*DSS1*	RNase, associates with the ribosome, turnover of aberrant RNAs
*YNL073w*	*MSK1*	Lysyl-tRNA synthetase, mitochondrial
*YOL033w*	*MSE1*	Glutamyl-tRNA synthetase, mitochondrial
*YOR065w	*CYT1*	Cytochrome c_1_
*YOR187w*	*TUF1*	Translation elongation factor Tu, mitochondrial
*YPL097w*	*MSY1*	Tyrosyl-tRNA synthetase, mitochondrial
*YPL104w*	*MSD1*	Aspartyl-tRNA synthetase, mitochondrial
*YPL148c	*PPT2*	Activates mitochondrial acyl carrier protein
*YPL254w	*HFI1*	Component of the ADA complex
*YPL271w	*ATP15*	Epsilon subunit of F_1_-ATP synthase
		
**ORFs encoding unknown proteins**		
*YDR065w	*RRG1*	Unknown function, protein is detected in highly purified mitochondria
*YDR114c		Dubious ORF, overlaps with *YDR115w*
*YGR102c		Unknown function, protein is detected in highly purified mitochondria
*YGR150c	*RRG2*	Unknown function, protein is detected in highly purified mitochondria
*YMR293c	*RRG6*	Unknown function, protein is detected in highly purified mitochondria
*YNL184c		Dubious ORF unlikely to encode a protein
*YPR116w	*RRG8*	Unknown function, GFP-tagged protein in mitochondria

The list indicates systematic and standard names of genes required for protein translation activity in class I and III *pet *mutants. The cellular roles of the proteins are indicated according to the Saccharomyces Genome Database (SGD) [19] or manually annotated. The list of genes has been matched to entries in SGD (biological process term: translation and cellular component term: mitochondrion). Genes that were previously not known to be required for mitochondrial translation are indicated with an asterisk.

**Figure 4:**
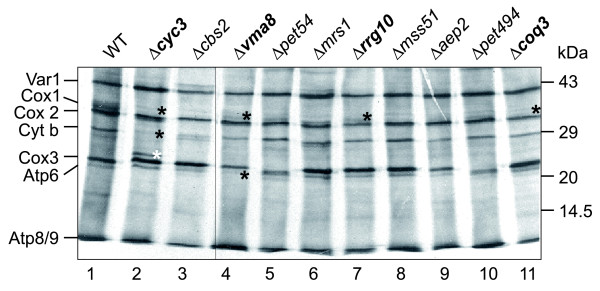
Mitochondrial protein synthesis in *pet *mutants showing an altered translation pattern. Yeast strains were grown in raffinose-containing minimal medium to logarithmic growth phase, cytosolic translation was stopped by the addition of cycloheximide, and newly synthesized mitochondrial proteins were labeled by the addition of ^35^S methionine. After an incubation of 30 minutes at 30°C, labeling of mitochondrial proteins was stopped by the addition of cold methionine and chloramphenicol, and cell extracts were analyzed by SDS-PAGE, transfer of proteins to nitrocellulose and autoradiography. All mutants have been analyzed in at least three independent experiments. The samples shown here have all been analyzed on the same gel (one lane has been spliced out as indicated by the thin line between lanes 3 and 4). For each strain the same amount of total cellular protein has been loaded per lane. Mutants that were previously not known to be affected in the synthesis of specific mitochondria-encoded proteins are in bold letters. Alterations of the translation pattern mentioned in the text are marked with asterisks. Black asterisks mark bands that are absent, and the white asterisk marks an additional band present in Δ*cyc3*. WT, wild type.

Specific alterations of the pattern of newly translated mitochondrial proteins were observed in ten mutants (Figure 4 and Table 4). A role in the expression of specific mitochondria-synthesized proteins has already been described for Aep2 [44], Cbs2 [45], Mrs1 [46], Mss51 [47], Pet54 [48,49], and Pet494 [50]. We observed that the pattern of mitochondrial translation products was also altered in Δ*coq3*, Δ*cyc3*, Δ*rrg10*, and Δ*vma8 *mutants. Coq3 is required for the biosynthesis of ubiquinone (coenzyme Q) in mitochondria [51]. We observed that mutant cells show a strong reduction of Cox1 (Figure 4, lane 11). Cyc3 is the mitochondrial cytochrome *c *heme lyase that attaches the heme cofactor to apo-cytochrome *c *in the intermembrane space [52]. Strikingly, mutant mitochondria show a strong reduction of Cox1 and cytochrome *b *and generate an additional protein band above Cox3 (Figure 4, lane 2), pointing to a role of Cyc3 also in the biogenesis of other mitochondrial proteins. Rrg10 is an uncharacterized mitochondrial protein that might play a specific role in the expression of the mitochondrial *COX1 *gene (Figure 4, lane 7), as discussed below. Cox1 and Atp6 are also reduced in the Δ*vma8 *mutant lacking a subunit of the vacuolar H^+ ^ATPase [53], suggesting that expression of these proteins is particularly sensitive to changes in cell metabolism (Figure 4, lane 4).

**Table 4:** Genes required for expression of specific mitochondrial translation products

*YAL039c	*CYC3*	Cytochrome *c *heme lyase
*YDR197c*	*CBS2*	Mitochondrial translational activator of the *COB *mRNA
*YEL051w	*VMA8*	Subunit D of the vacuolar H^+^-ATPase (V-ATPase)
*YGR222w*	*PET54*	Binds to the 5' untranslated region of the *COX3 *mRNA to activate its translation; also binds to the *COX1 *group I intron AI5 beta to facilitate splicing
*YIR021w*	*MRS1*	Required for the splicing of two mitochondrial group I introns
*YJL062w-a	*RRG10*	Protein of unknown function
*YLR203c*	*MSS51*	Required for translation of *COX1 *mRNA
*YMR282c*	*AEP2*	Likely involved in translation of the mitochondrial *ATP9 *mRNA
*YNR045w*	*PET494*	Mitochondrial translational activator specific for the *COX3 *mRNA
*YOL096c	*COQ3*	Component of a mitochondrial ubiquinone-synthesizing complex

The list indicates systematic and standard names of genes required for synthesis of only a subset of mitochondria-encoded proteins. The cellular roles of the proteins are indicated according to the Saccharomyces Genome Database [19] or manually annotated. Genes that were previously not known to be required for expression of specific mitochondrial translation products are highlighted with an asterisk.

### Other genes important for respiration

In sum, 61 respiratory-deficient mutants showed a wild-type-like mitochondrial translation pattern (Additional data file 8). We conclude that these genes are not essential for mitochondrial genome maintenance or mitochondrial protein synthesis. This group contains 32 genes encoding known mitochondrial proteins, many of which are required for assembly of the respiratory chain. Eighteen genes encode known extra-mitochondrial proteins, and 11 ORFs are uncharacterized. Five of the uncharacterized ORFs are unlikely to encode proteins because they overlap with known protein-coding genes, whereas six ORFs (*YDL129w*, *YDL133w*, *YDL033w*/*RRG4*, *YNL213c*/*RRG9*, *YOL071w*, and *YOL083w*) might encode novel proteins involved in maintenance of respiratory activity. Possible roles of Rrg4 and Rrg9 in this process are discussed below.

Half of the *pet *genes encoding extra-mitochondrial proteins are associated with vacuolar functions (Additional data file 8). Moreover, a surprisingly large number of genes encoding V-ATPase subunits are highly penetrant *pet *genes (Figure 1c; Additional data file 3). What might be the function of the vacuole in maintenance of respiratory activity in yeast? We suggest three possibilities. First, vacuolar functions in metabolite storage or in cytosolic ion and pH homeostasis [54,55] might interfere with mitochondrial metabolism. Second, loss of V-ATPase activity has been reported to render cells hypersensitive to oxidative stress [56-58], which might have an impact on mitochondrial functions as well. And third, the vacuole is the terminal compartment receiving cellular components destined for degradation by autophagic pathways. As also mitochondria are degraded by autophagy in yeast [59], it is possible that the vacuole plays an important role in mitochondrial quality control and turn-over. The high number of *pet *mutants lacking V-ATPase subunits clearly demonstrates that there is an important - as yet not fully understood - functional relationship between the vacuole and mitochondria.

### Contribution of acquired defects to maintenance of respiratory activity

The respiratory-deficient phenotype of 23 *pet *mutants was rescued by mating with Δ*mip1 *as well as by cytoduction (class II; Additional data file 9). These mutants contained a [*rho*^+^] mitochondrial genome, as indicated by the mating experiment. In addition, three independently performed cytoduction experiments suggest that replenishment of cytoplasmic material reproducibly restores and maintains respiratory growth, at least for a few generations. These observations point to the possibility that respiratory competence may involve acquired properties that are not strictly linked to the nuclear or mitochondrial genotype. In order to corroborate this assumption, we tested whether cytoduction with a [*rho*^0^] donor strain would also restore respiratory growth. Rescue was observed in 11 strains (Additional data file 9), suggesting that, at least in some cases, cytoplasmic components other than mtDNA are able to improve respiratory functions. We hypothesize that respiratory deficiency may be an acquired phenotype that does not exclusively depend on the genotype.

**Figure 5:**
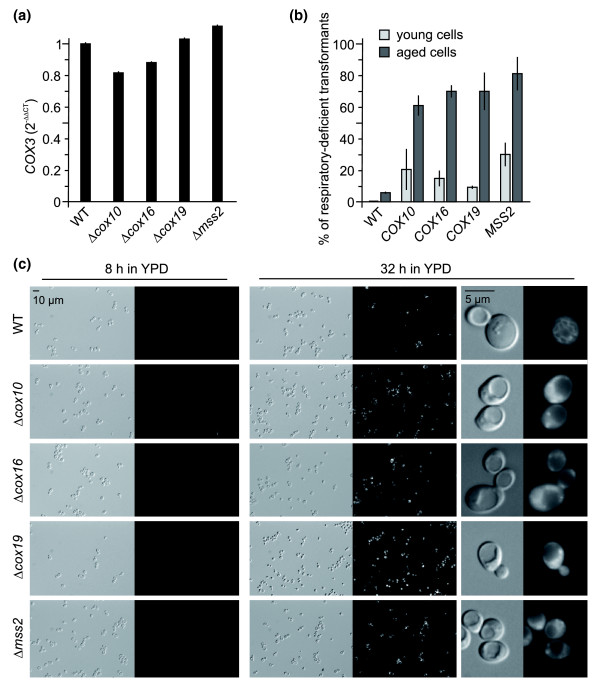
Acquired phenotypes of COX assembly mutants. **(a) **Quantification of mtDNA. Yeast strains were grown overnight in liquid glucose-containing medium. Total DNA was isolated and the copy number of the mitochondrial *COX3 *gene was related to that of the nuclear *GAL4 *gene by RT-PCR and calculation of the 2^-ΔΔ*C*^_T _value. Error bars indicate standard deviations of triplicate PCR reactions. **(b) **Complementation test. Δ*cox10*, Δ*cox16*, Δ*cox19*, and Δ*mss2 *strains taken from the *MAT*α yeast deletion library have been transformed with single copy plasmids carrying the respective complementing wild-type alleles under control of their endogenous promoters. Wild-type cells (WT) were transformed with an empty vector. Young cells were grown on complete medium at 30°C overnight before transformation (light bars). Aged cells were incubated on complete medium at room temperature for 14 to 28 days before they were transferred to fresh plates, grown at 30°C overnight, and transformed with complementing plasmids (dark bars). Three days after transformation, colonies were replicated on plates containing fermentable or non-fermentable carbon sources, and the percentage of respiratory-deficient transformants was determined. Error bars indicate standard deviations of three independent experiments. **(c) **ROS accumulation. Yeast strains were grown for the indicated time periods in liquid glucose-containing medium (YPD), stained by the addition of DHR and analyzed by differential interference microscopy (left panels) and fluorescence microscopy (right panels). All fluorescent micrographs were taken with identical camera settings.

Among ten class II mutants lacking known mitochondrial proteins (Δ*coq5*, Δ*coq10*, Δ*cox10*, Δ*cox16*, Δ*cox19*, Δ*mct1*, Δ*mss2*, Δ*nfu1*, Δ*slm3*, and Δ*som1*) are four mutants that are specifically defective in the assembly of the cytochrome *c *oxidase (COX complex). Cox10 is required for the synthesis of the heme A cofactor [60,61], Cox19 is a metallochaperone that delivers copper to the COX complex [62], Mss2 is required for the membrane translocation of the carboxyl terminus of the mitochondria-encoded Cox2 protein [63], and Cox16 contributes to assembly of the COX complex by an as yet unknown mechanism [64]. Intriguingly, all four of these proteins are required for assembly of COX subunits at a post-translational stage. While respiratory-deficiency in Δ*cox10*, Δ*cox16*, Δ*cox19*, and Δ*mss2 *mutants has been documented before [60,62-64], we asked whether acquired properties might contribute to the loss of respiratory activity in these mutants. To exclude effects due to differences in mtDNA copy number, we first quantified the abundance of the mitochondrial *COX3 *gene by RT-PCR. We found that mtDNA is stably maintained in Δ*cox10*, Δ*cox16*, Δ*cox19*, and Δ*mss2 *mutants at a level very similar to wild-type cells (Figure 5a).

Next, we tried to rescue the deletion mutants with plasmids encoding wild-type copies of the respective genes under control of their endogenous promoters. Remarkably, after growth on selective medium a substantial number of transformants remained respiratory-defective after complementation with the respective wild-type gene (Figure 5b). The occurrence of respiratory-deficient clones was not induced by the transformation procedure *per se *because transformation of wild-type cells with the same plasmids yielded 100% respiration-competent clones (not shown). In order to test whether Δ*cox10*, Δ*cox16*, Δ*cox19*, and Δ*mss2 *clones lose properties required for respiratory competence over time, we subjected the deletion mutants to chronological aging [65], that is, continued incubation of stationary phase cultures. Mutant cells were incubated on glucose-containing medium for several days at room temperature before transformation with the complementing plasmids. Under these conditions, the fraction of clones that could not be rescued increased to 60 to 81% for mutant cells, whereas only 6% of aged wild-type clones were observed to be respiratory-deficient after transformation (Figure 5b). This suggests that mitochondria in Δ*cox10*, Δ*cox16*, Δ*cox19*, and Δ*mss2 *cells become irreversibly damaged over time, producing a respiratory-deficient phenotype that cannot be rescued any more. Apparently, this damage is already induced during vegetative growth and is markedly enhanced during aging.

As mitochondrial metabolism and aging are linked to the generation of potentially harmful reactive oxygen species (ROS) [66] we asked whether ROS accumulate in COX assembly mutants. High levels of ROS generated in yeast cells convert the non-fluorescent compound dihydrorhodamine 123 (DHR) to the oxidized fluorescent chromophore rhodamine 123 [67]. Upon incubation of young wild-type Δ*cox10*, Δ*cox16*, Δ*cox19*, and Δ*mss2 *cultures with DHR (8 h in liquid YPD medium) only very few cells showed significant staining (Figure 5c). After continued incubation (32 h), about 60% of wild-type cells and 90 to 98% of mutant cells showed significant rhodamine staining (Figure 5c). Very similar results were obtained when aging was allowed for up to 80 h (not shown). Furthermore, we noticed that rhodamine staining in wild-type cells was relatively faint and often restricted to tubular structures (presumably representing the mitochondrial network), whereas the signal was much stronger and dispersed throughout the cytosol in mutant cells (Figure 5c). We conclude that Δ*cox10*, Δ*cox16*, Δ*cox19*, and Δ*mss2 *cells produce elevated ROS levels during chronological aging. Presumably, ROS induce irreversible damage to mitochondrial proteins, lipids and/or mtDNA, thereby preventing rescue of the mutant phenotype by transformation with complementing plasmids. On the other hand, replenishment of fresh mitochondria by cytoduction might improve respiratory performance, at least for a limited time. It remains to be shown whether accumulation of ROS-induced damage is a general feature of class II mutants.

### Novel components essential for respiratory growth

All previously uncharacterized *RRG *genes analyzed herein can be clearly related to mitochondrial functions. Proteins Rrg1, Rrg2, and Rrg5 through Rrg10 have been localized to mitochondria by high-throughput green fluorescent protein (GFP) fusion protein localization [35] and/or mitochondrial proteome analysis [6,34]. The Rrg3 protein carries a putative mitochondrial presequence, whereas the intracellular location of Rrg4 remains unknown. Functional properties of *RRG *genes are summarized in Table 5.

**Table 5:** Functional properties of newly described *RRG *genes

	**Systematic name**	**Mitochondrial localization**	**Δ*mip1 *mating**	**Cytoduction**	**Nucleoids after cytoduction**	**Translation activity after cytoduction**
*RRG1*	*YDR065w*	[34]	-	-	Altered	Absent
*RRG2*	*YGR150c*	[34,35]	-	-	Altered	Absent
*RRG3 (AIM22)*	*YJL046w*	[69]	+	-	WT	WT
*RRG4 (IRC19)*	*YLL033w*	Unknown	-	-	WT	WT
*RRG5 (GEP5)*	*YLR091w*	[6,34,35]	-	-	Absent	Absent
*RRG6 (HER2)*	*YMR293c*	[6,34,35]	-	-	Altered	Absent
*RRG7*	*YOR305w*	[35]	+	+	ND	ND
*RRG8*	*YPR116w*	[35]	-	-	Altered	Absent
*RRG9*	*YNL213c*	[34]	-	-	Altered	WT
*RRG10*	*YJL062w-a*	[34,35]	+	-	WT	Altered

References refer to published evidence of mitochondrial localization of Rrg proteins; + indicates rescue by mating with Δ*mip1 *or cytoduction, respectively. WT, wild type-like; ND, not determined. See text for details.

Δ*rrg1*, Δ*rrg2*, Δ*rrg4*, Δ*rrg5*, Δ*rrg6*, Δ*rrg8*, and Δ*rrg9 *are class I *pet *mutants lacking a functional mitochondrial genome. DAPI staining revealed defects in the organization of mtDNA that emerged early after introduction of wild-type mitochondrial genomes by cytoduction in Δ*rrg1*, Δ*rrg2*, Δ*rrg6*, Δ*rrg8*, and Δ*rrg9 *mutants. Nucleoids appeared larger compared to the wild type, the number of nucleoids per cell was reduced, and several cells were completely devoid of mtDNA (not shown). These observations suggest that Rrg1, Rrg2, Rrg6, Rrg8, and Rrg9 play an important role in maintenance of mtDNA. Immediate and complete loss of mtDNA after cytoduction in the Δ*rrg5 *mutant indicates an essential role of Rrg5 for maintenance of mtDNA (see above).

Interestingly, Rrg2 contains a pentatricopeptide (PPR) motif. PPR protein-encoding genes can be found in virtually all sequenced eukaryotic genomes, but are particularly abundant in plants. PPR proteins are localized in plastids and mitochondria where they are involved in the control of various stages of gene expression [68]. Lack of mitochondrial translation activity and early loss of mtDNA observed here are consistent with a role of Rrg2 in control of mitochondrial gene expression.

Δ*rrg3 *is a class III *pet *mutant able to maintain a [*rho*^+^] genome and wild-type-like mitochondrial protein translation activity. Although a mitochondrial location of Rrg3 has not been shown experimentally, the Mitoprot program [69] predicts the presence of a mitochondrial presequence with a high probability (0.9484). Mutants lacking Rrg3 (alternative name Aim22) show an increased *petite *frequency [70]. The protein has high homology to lipoate-protein ligase A family members [71]. Thus, it is conceivable that Rrg3 mediates the attachment of the lipoic acid cofactor to mitochondrial multienzyme complexes, such as pyruvate dehydrogenase, α-ketoglutarate dehydrogenase, glycine decarboxylase or others. Intriguingly, it has recently been reported that lipoate-protein ligase activity is important for maturation of RNase P, an enzyme that processes mitochondrial precursor tRNAs [72]. It will be interesting to determine whether Rrg3 plays a specific role in this process.

The Δ*rrg4 *mutant has recently been identified as one of 86 gene deletion mutants that show an increased assembly of Rad52, a central protein of the homologous recombination machinery, in subnuclear foci reflecting DNA repair centers. Therefore, the gene has been named *IRC19 *(for 'increased recombination centers') [73]. Interestingly, several other genes related to mitochondrial function were also isolated in this screen, including *CBT1*, *COX16*, *MRP17*, *MRPL1*, *MRPS16*, and *YMR31*. It has been suggested that an increase of oxidative damage due to impaired respiratory chain functions might stimulate spontaneous DNA lesions in the nucleus and, therefore, constitutes a functional link between mitochondrial respiration and DNA repair processes in the nucleus [73]. As a Rrg4-GFP fusion protein can not be visualized in cells [35], the intracellular location of Rrg4 remains unknown.

The *RRG6 *gene has recently been found in a screen for components involved in remodelling of the endoplasmic reticulum (ER). It has been named *HER2 *(Hmg2-induced ER remodelling); however, its molecular role in shaping the ER membrane remained unknown [74]. As the Rrg6 protein has been localized to mitochondria by both GFP tagging and proteome analysis [6,34,35], we propose that its primary function is related to maintenance of respiratory activity. The protein is highly homologous to bacterial glutamyl-tRNA amidotransferases, and a role in mitochondrial protein synthesis is consistent with our observation that mitochondrial translation is blocked in the Δ*rrg6 *mutant (Table 3). Recently, *RRG5 *(alternative name *GEP5*) and *RRG6 *(alternative names *GEP6 *or *HER2*) have been shown to genetically interact with genes encoding prohibitin ring complexes in the mitochondrial inner membrane [75]; however, the functional significance of this interaction is not yet understood.

Δ*rrg7 *is a class II *pet *mutant presumably acquiring respiratory deficiency independent of its genotype. The *RRG7 *gene encodes a mitochondrial protein [35] that has homologs in fungi and other lower eukaryotes. Its function in mitochondrial biogenesis is currently unknown; however, the deletion mutant has been reported to exhibit increased sensitivity to the synthetic tripeptide arsenical 4-(*N*-(*S-*glutathionylacetyl)amino) phenylarsenoxide that targets mitochondria by inactivating the adenine nucleotide translocator. This drug inhibits proliferation of actively dividing endothelial cells and is an inhibitor of angiogenesis during tumor formation [76].

Δ*rrg10 *is a class III *pet *mutant able to maintain a [*rho*^+^] genome. The *RRG10 *gene encodes a small mitochondrial protein [34,35] of only 85 amino acid residues. Analysis of the mitochondrial translation pattern revealed a reduction of Cox1, suggesting that Rrg10 plays a specific role in transcription or maturation of mitochondrial mRNAs and/or translation or assembly of mitochondrial gene products.

## Conclusions

Surprisingly, only a limited number of mutants reproducibly show a *pet *phenotype when different versions of the yeast deletion library are screened for growth on non-fermentable carbon sources. While some differences can be ascribed to wrong deletions present in the library, most of the variations are likely due to intrinsic properties of the mutant strains. We present four lines of evidence suggesting that the plasticity of *pet *phenotypes is much greater than previously anticipated. First, several deletions produce different phenotypes in different versions of the deletion library (Figure 1a, b; Additional data file 3); second, a number of mutants lose mtDNA at a high rate upon continued incubation in glucose-containing medium (Additional data file 6); third, respiratory deficiency can be reversed by relief of catabolite repression in a relatively large number of mutants (Additional data file 7); and fourth, several [*rho*^+^] *pet *mutants accumulate irreversible damage resulting in an improvement of respiratory performance after cytoduction (Figure 5; Additional data file 9). It is a challenge for the future to examine further contributions of environmental factors, nutrient supply, and possible epigenetic mechanisms to phenotypic plasticity.

Comparative gene deletion analysis enabled us to define by stringent criteria a set of 163 protein-coding genes (13 dubious ORFs subtracted from the 176 mutants found in all *pet *screens of the library) that are obligatorily required for respiratory metabolism in yeast. These include ten largely uncharacterized genes, *RRG1 *through *RRG10*. Remarkably, almost all of these highly penetrant mutants (95%) have been reported to show decreased fitness on non-fermentable carbon sources when the whole-genome pool of yeast deletion mutants was analyzed [77,78]. While the approach pursued by Steinmetz *et al*. [77] resulted in a relatively large set of genes potentially required for respiratory growth (466 genes, 43.1% of which encode mitochondrial proteins), the comparative gene deletion approach pursued here apparently is more selective (176 genes, 73.3% of which encode mitochondrial proteins). A high resolution of our comparative gene deletion analysis is also apparent from a comparison with the results we obtained after our first screen of the deletion library reported in the Dimmer *et al*. study [14], which yielded 341 *pet *genes (only 54.8% of which encode mitochondrial proteins). Our present work suggests that 165 of these originally identified mutants do not reproducibly give rise to a *pet *phenotype and should be considered as important but not essential for respiratory growth of yeast. Thus, Figure 1c gives a significantly improved representation of cellular functions of genes essential for respiration. A recent study by Hess *et al*. [70] reports a computational prediction of 193 candidate genes and subsequent analysis of their possible roles in mitochondrial biogenesis. They found that Δ*rrg2 *and Δ*rrg6 *mutants are respiratory deficient, and that the Δ*rrg3 *mutant shows an increased *petite *frequency [70]. However, the remaining seven *RRG *genes were only found by the comparative gene deletion analysis described here, demonstrating the value of our approach.

The systematic functional analysis of *pet *mutants reported here uncovered roles of 8 novel components in mtDNA maintenance, 30 novel components in mitochondrial protein synthesis, and 4 novel components in expression of specific mitochondrial translation products. We suggest that these data may serve as positive lists for genes important for respiratory growth, mtDNA maintenance and mitochondrial protein synthesis. It should be pointed out that components might have been missed that are encoded by redundant genes or that are not correct in the deletion library. Furthermore, some genes might be specifically required only under certain growth conditions or in certain genetic backgrounds. While a mechanistic understanding of the molecular processes contributing to respiratory activity will require further rigorous experimentation, the systematic large-scale functional analysis of *pet *mutants reported here is a first step towards a definition of the complements of genes required for maintenance of the mitochondrial genome and mitochondrial protein translation. Together with integrated analyses of different genomic and proteomic approaches [78], combination of computational approaches with quantitative experimentation [70], and the construction of protein interaction networks [79] it will contribute to an understanding of the systems properties of mitochondria with steadily increasing resolution.

## Materials and methods

### Yeast strains and plasmids

Yeast strains used in this study were isogenic to BY4741, BY4742 and BY4743 [18], with the exception of strain J1361 [80], which was used for cytoduction. The [*rho*^0^] cytoduction donor strain was generated by growth of J1361 overnight in YPD medium supplemented with 50 μg/ml ethidium bromide. Complete loss of mtDNA was controlled by DAPI staining. The *MAT*α gene deletion library [16] and its supplement covering newly assigned small ORFs [21] was obtained from BioCat (Heidelberg, Germany), and *MAT*a single mutant Δ*mip1 *was obtained from EUROSCARF (Frankfurt, Germany). Plasmid pRS416/*MSS2 *was constructed by PCR amplification of the *MSS2 *gene using primers 5' AAA GGA TCC GAT TTT ATG TGT GGA ATG CTA ACG ATG AAC and 5' AAA CTC GAG CTC TAA CAG TAT TTC CTA ATT ATT TCA TAG GTA AC and subcloning into the *Bam*HI and *Xho*I sites of vector pRS416 [81]. Plasmid pRS416/*COX16 *was constructed by PCR amplification of the *COX16 *gene using primers 5' AAA GGA TCC AAT ATT ACC GTG AAT ATC GCG AGC TAC and 5' AAA CTC GAG AGG TAT TTA CAA TCA TTT CCT AGA CAT TCT and subcloning into the *Bam*HI and *Xho*I sites of vector pRS416. For complementation tests, yeast strains were transformed with plasmids pG12/T4 [60] expressing *COX10*, pRS416/*COX16*, pG188/T1 [62] expressing *COX19*, or pRS416/*MSS2*.

PCR analyses to confirm the identity of deletion mutants were performed in a way that one primer was homologous to a sequence within the coding region or within the deletion marker cassette, respectively, whereas the other primer was homologous to a sequence outside the deleted part of the gene. Thus, a PCR product can be generated only if the correct allele corresponding to the primer combination is present. Primers used to confirm the identity of yeast deletion mutants are listed in Additional data file 10.

### Yeast genetic methods

*S. cerevisiae *was cultivated and manipulated according to standard procedures [82]. For screening for respiratory-deficient mutants, yeast deletion strains were manually transferred with a sterile pinning tool from 96-well plates to rich media plates with either 2% glucose as fermentable (YPD) or 3% glycerol as non-fermentable (YPG) carbon source. The screening of the entire library on YPG was performed once. The screening procedure was as similar as possible to the screen performed earlier by us in the Dimmer *et al*. study [14]. Respiratory-deficient mutants were screened in addition on media containing 3% ethanol or 3% lactate (pH adjusted to 7.0 with NaOH) as non-fermentable carbon sources. The growth behavior was evaluated by visual inspection after 3 days (YPD) or 6 days (YPG and other non-fermentable media) of incubation at 30°C.

For high-throughput complementation tests with Δ*mip1*, yeast deletion strains were transferred with a sterile pinning tool from 96-well plates to a lawn of *MAT*a Δ*mip1 *cells on YPD plates. After incubation overnight to allow for mating, cells were replica-plated two times on plates containing minimal SD medium selective for diploid cells. Then, growth on YPG plates was determined as above. Cytoduction was performed as described [80]. Cytoduction experiments were repeated at least three times. To adapt yeast deletion strains to non-fermentable carbon sources, cells were transferred from YPD plates to YPG plates containing 0.1% glucose, replica-plated once on YPG/0.1% glucose and then replica-plated on YPG.

### Analysis of mitochondrial translation products

Labeling of mitochondrial translation products *in vivo *was performed essentially as described [83] with the following minor modifications: cytosolic translation was stopped with 0.3 mg/ml cycloheximide, the labeling reaction was performed for 30 minutes, and the chase reaction was performed for 15 minutes. Mitochondrial translation products were analyzed by SDS-PAGE, transfer to nitrocellulose and autoradiography.

### Assay of accumulation of irreversible damage during chronological aging

Using sterile toothpicks, cells were taken from glycerol stocks and spread on YPD plates as patches of about 2 cm^2 ^size. Plates were incubated for 14 to 28 days at room temperature to allow chronological aging. Then, a small amount of aged cells (or young cells taken directly from glycerol stocks as a control) was spread on a fresh YPD plate, incubated overnight at 30°C, and transformed according to the rapid transformation protocol described by Truong and Gietz [84]. Using sterile toothpicks, at least 100 transformants were transferred as short streaks (approximately 1 cm) to fresh SD plates selective for the marker of the transformed plasmids. Plates were incubated for 1 day at 30°C and then replica-plated on YPD and YPG using sterile velvet. Carbon source-dependent growth of transformants was visually scored after 2 to 3 days at 30°C.

### Staining of mtDNA, DHR staining and microscopy

Staining of mtDNA with DAPI in methanol-fixed cells was as described [30]. For the analysis of ROS production, 1 μl DHR (2.5 mg/ml in DMSO) was added to 500 μl cell suspension and incubated for 2 h at 30°C. Cells were harvested by centrifugation, washed in phosphate-buffered saline, resuspended in phosphate-buffered saline and analyzed by microscopy. Epifluorescence microscopy was performed using a Zeiss Axioplan 2 microscope equipped with a HBO 100 mercury lamp, Zeiss filter sets 01 and 09 and a Plan-Neofluar 100× 1.30 NA Ph3 oil objective (Carl Zeiss Lichtmikroskopie, Göttingen, Germany). Images were recorded with an Evolution VF Mono Cooled monochrome camera (Intas, Göttingen, Germany) and processed with Image Pro Plus 5.0 and Scope Pro 4.5 software (MediaCybernetics, Silver Springs, MD, USA).

### RT-PCR

Yeast strains were grown overnight in liquid YPD medium and DNA was extracted using the YeaStar™ Genomic DNA Kit (Zymo Research, Orange, USA) according to the manufacturer's instructions. PCR reactions were performed in 20 μl volume in 96-well plates using Maxima™ SYBR Green qPCR Master Mix (2×) (Fermentas, St Leon-Rot, Germany) according to the manufacturer's instructions in an ABI PRISM 7000 Sequence Detection System (Applied Biosystems, Foster City, CA, USA). The following primers were used: *GAL4 *forward, 5' TTT CTC CTG GCT CAG TAG GGC; *GAL4 *reverse, 5' AGT TAC GAG AGG GTG GAC GGT; *COX3 *forward, 5' ATT GAA GCT GTA CAA CCT ACC GAA TT; *COX3 *reverse, 5' CCT GCG ATT AAG GCA TGA TGA. Data were analyzed with Sequence Detection Software Version 1.2.3 7000 System SDS software Core Application (Applied Biosystems) and calculated according to the 2^-ΔΔ*C*^_T_-method [85].

## Abbreviations

COX: cytochrome *c *oxidase; DAPI: 4',6-diamidino-2-phenylindole; DHR: dihydrorhodamine 123; ER: endoplasmic reticulum; GFP: green fluorescent protein; mtDNA: mitochondrial DNA; ORF: open reading frame; PPR: pentatricopeptide; ROS: reactive oxygen species.

## Authors' contributions

SM performed the experiments, SM and BW conceived the study, analyzed the data and wrote the manuscript.

## Additional data files

The following additional data are available with the online version of this paper: a table listing *pet *genes isolated from the *MAT*a deletion library (Additional data file 1); a table listing *pet *genes unique to this study (Additional data file 2); a table listing *pet *genes grouped according to their occurrence in *pet *screens and localization and function of the encoded gene products (Additional data file 3); a table listing *pet *genes producing growth defects only on specific carbon sources (Additional data file 4); a table listing mutants belonging to four classes of *pet *genes (Additional data file 5); a table showing quantification of loss of mtDNA in class IV *pet *mutants (Additional data file 6); a table listing *pet *genes dispensable for respiration (Additional data file 7); a table listing genes required for respiratory activity in class I and III *pet *mutants that show a wild-type pattern of mitochondrial translation products (Additional data file 8); a table listing genes possibly affecting mitochondrial function in combination with acquired defects (Additional data file 9); a table listing primers used to confirm the identity of yeast deletion mutants (Additional data file 10).

## Supplementary Material

Additional data file 1 *pet *genes isolated from the *MAT*α deletion library. Click here for file

Additional data file 2 *pet *genes unique to this study. Click here for file

Additional data file 3 *pet *genes grouped according to their occurrence in *pet *screens and localization and function of the encoded gene products. Click here for file

Additional data file 4 *pet *genes producing growth defects only on specific carbon sources. Click here for file

Additional data file 5 Mutants belonging to four classes of *pet *genes. Click here for file

Additional data file 6 Quantification of loss of mtDNA in class IV *pet *mutants. Click here for file

Additional data file 7 *pet *genes dispensable for respiration. Click here for file

Additional data file 8 Genes required for respiratory activity in class I and III *pet *mutants that show a wild-type pattern of mitochondrial translation products. Click here for file

Additional data file 9 Genes possibly affecting mitochondrial function in combination with acquired defects. Click here for file

Additional data file 10 Primers used to confirm the identity of yeast deletion mutants. Click here for file
