# Supplementary material for: Genome-wide deletion mutant analysis reveals genes required for respiratory growth, mitochondrial genome maintenance and mitochondrial protein synthesis in Saccharomyces cerevisiae
Source: Genome Biol. 2009 Sep 14;10(9):R95. doi: 10.1186/gb-2009-10-9-r95 (PMC2768984; doi:10.1186/gb-2009-10-9-r95)
Supplement: Additional data file 1 — pet genes isolated from the MATα deletion library. [file gb-2009-10-9-r95-S1.PDF]

Supplemental table 1. *pet* genes isolated from the *MAT*α deletion library. The list indicates systematic and standard names and a brief description of gene function according to the Saccharomyces Genome Database and manual annotation.

YAL009W	SPO7, ER membrane protein of unknown function
YAL010C	MDM10, involved in mitochondrial morphology and inheritance
YAL013W	DEP1, transcriptional modulator
YAL026C	DRS2, maintains membrane lipid asymmetry in post-Golgi secretory vesicles
YAL039C	CYC3, holocytochrome c synthase (cytochrome c heme lyase)
YAL044C	GCV3, glycine decarboxylase hydrogen carrier protein H subunit
YBL019W	APN2, Class II abasic (AP) endonuclease; repair of DNA damage; homolog of hHAP1
YBL021C	HAP3, component of heterotrimeric CCAAT-binding factor
YBL031W	SHE1 Cytoskeletal protein of unknown function; overexpression causes growth arrest
YBL032W	HEK2, RNA binding protein; localizes ASH1 mRNA
YBL036C	non-specific single-domain racemase
YBL045C	COR1, ubiquinol cytochrome c reductase core protein 1
YBL046W	PSY4, regulatory subunit of a protein phosphatase complex (nuclear)
YBL053W	Dubious ORF, overlaps with SAS3n
YBL057C	PTH2, negatively regulates the ubiquitin-proteasome pathway
YBL062W	Dubious ORF, overlaps with SKT5
YBL080C	PET112, Protein required for mitochondrial translation
YBL082C	ALG3, alpha(1-3) mannosyltransferase
YBL090W	MRP21, mitochondrial ribosomal protein
YBL093C	ROX3, RNA polymerase II holoenzyme component
YBL099W	ATP1, alpha subunit of F1-ATP synthase
YBL100C	Dubious ORF, overlaps with ATP1
YBR003W	COQ1, hexaprenyl pyrophosphate synthetase
YBR026C	ETR1, localized to in mitochondria, where it has a probable role in fatty acid synthesis
YBR039W	ATP3, Gamma subunit of the F1 sector of mitochondrial F1F0 ATP synthase
YBR097W	VPS15, serine/threonine protein kinase involved in vacuolar protein sorting
YBR128C	ATG14, Subunit of an autophagy-specific phosphatidylinositol 3-kinase complex
YBR146W	MRPS9, Mitochondrial ribosomal protein of the small subunit
YBR179C	FZO1, transmembrane GTPase required for mitochondrial fusion
YBR251W	MRPS5, mitochondrial ribosomal protein
YBR268W	MRPL37, mitochondrial ribosomal protein
YBR282W	MRPL27, mitochondrial ribosomal protein
YBR283C	SSH1, involved in co-translational protein translocation in the ER
YBR289W	SNF5, component of SWI-SNF global transcription activator complex
YCL001W-A	Unknown function
YCL007C	Dubious ORF; overlaps verified ORF YCL005W-A
YCL010C	SGF29, Probable 29kKDa Subunit of SAGA histone acetyltransferase complex
YCR003W	MRPL32, mitochondrial ribosomal protein
YCR020W-B	HTL1, Subunit of the RSC chromatin remodeling complex
YCR024C	SLM5, Asparaginyl-tRNA synthetase, mitochondrial
YCR028C-A	RIM1, binds single-stranded DNA, required for DNA replication in mitochondria
YCR046C	IMG1, Mitochondrial ribosomal protein of the large subunit
YCR071C	IMG2, Mitochondrial ribosomal protein of the small subunit
YDL012C	Plasma membrane protein of unknown function
YDL033C	SLM3, tRNA-specific 2-thiouridylase
YDL039C	PRM7, Pheromone-regulated protein
YDL044C	MTF2, mitochondrial protein involved in mRNA splicing and protein synthesis
YDL045W-A	MRP10, mitochondrial ribosomal protein
YDL056W	MBP1, transcription factor that collaborates with Swi6p
YDL067C	COX9, cytochrome c oxidase subunit VIIA
YDL068W	Dubious ORF, overlaps with CBS1
YDL077C	VAM6, Vacuolar protein, tethers steps of vacuolar membrane fusion

YDL091C	UBX3, UBX domain-containing protein that interacts with Cdc48p
YDL099W	BUG1, involved in ER to Golgi transport
YDL107W	MSS2, required for export of C-terminal tail of Cox2p through the inner membrane
YDL114W	Putative protein of unknown function with similarity to acyl-carrier-protein reductases
YDL129W	Unknown function
YDL133W	Unknown function
YDL157C	unknown function; detected in highly purified mitochondria in high-throughput studies
YDL185W	VMA1, catalytic subunit (subunit A) of the vacuolar H(+) ATPase V1 complex
YDL192W	ARF1, GTPase of the Ras superfamily involved in coated vesicles formation
YDR010C	Dubious ORF
YDR025W	RPS11A, Protein component of the small (40S) ribosomal subunit
YDR065W	Unknown function
YDR079W	PET100, required for assembly of cytochrome c oxidase
YDR114C	Dubious ORF, overlaps with YDR115W
YDR115W	Putative mitochondrial ribosomal protein
YDR116C	MRPL1, Mitochondrial ribosomal protein of the large subunit
YDR129C	SAC6, actin filament bundling protein, fimbrin; essential for polarized secretion
YDR148C	KGD2, 2-oxoglutarate dehydrogenase complex E2 component
YDR175C	RSM24, mitochondrial ribosomal protein
YDR194C	MSS116, mitochondrial RNA helicase, required for splicing of group II introns
YDR197W	CBS2, translational activator for cyt b
YDR204W	COQ4, involved in biosynthesis of coenzyme Q
YDR230W	Dubious ORF, overlaps with COX20
YDR237W	MRPL7, mitochondrial ribosomal protein
YDR269C	Dubious ORF, overlaps with CCC2
YDR270W	CCC2, copper-transporting P-type ATPase
YDR271C	Dubious ORF, overlaps with CCC2
YDR296W	MHR1, involved in repair, recombination and maintenance of mitochondrial DNA
YDR298C	ATP5, subunit 5 of F0-ATP synthase, oligomycin sensitivity-conferring subunit
YDR337W	MRPS28, mitochondrial ribosomal protein
YDR347W	MRP1, mitochondrial ribosomal protein
YDR350C	ATP22, required for assembly of the F0 sector of mitochondrial F1F0 ATP synthase
YDR364C	CDC40, pre-mRNA splicing factor
YDR375C	BCS1, required for expression of functional Rieske iron-sulfur protein
YDR377W	ATP17, ATP synthase subunit f
YDR448W	ADA2, component of the histone acetyltransferase complexes
YDR458C	HEH2, Protein of unknown function; GFP-fusion protein in nuclear periphery
YDR491C	Dubious ORF, overlaps with IZH1
YDR523C	SPS1, Putative protein serine/threonine kinase
YDR529C	QCR7, ubiquinol cytochrome c reductase subunit 7
YEL024W	RIP1, ubiquinol cytochrome c reductase iron-sulfur protein
YEL027W	CUP5, V-ATPase 16 kDa proteolipid subunit of membrane (V0) sector
YEL050C	RML2, mitochondrial ribosomal protein L2 of the large subunit
YEL051W	VMA8, vacuolar H(+)-ATPase (V-ATPase) subunit of the V1 catalytic sector
YEL059C-A	SOM1, Subunit of the mitochondrial inner membrane peptidase
YER014C-A	BUD25, involved in bipolar budding
YER017C	AFG3, involved in proteolytic and chaperone activities at the inner membrane
YER050C	RSM18, component of the mitochondrial ribosomal small subunit
YER061C	CEM1, beta-ketoacyl-ACP synthase, mitochondrial
YER070W	RNR1, ribonucleosid-diphosphate-reductase, large (R1) subunit
YER087W	similarity to tRNA synthetases; protein is detected in mitochondria
YER114C	BOI2, Protein implicated in polar growth, functionally redundant with Boi1p
YER131W	RPS26B, Protein component of the small (40S) ribosomal subunit
YER145C	FTR1, iron permease that mediates high-affinity iron uptake
YER154W	OXA1, component of the mitochondrial protein export machinery
YER155C	BEM2, Rho GTPase activating protein; control of cytoskeleton organization
YFL016C	MDJ1, DnaJ co-chaperone involved in mitochondrial biogenesis and protein folding
YGL017W	ATE1, Arginyl-tRNA-protein transferase
YGL070C	RPB9, RNA polymerase II, non-essential subunit, not shared

YGL071W	RCS1, transcription factor regulates genes involved in iron uptake and cell size
YGL129C	RSM23, mitochondrial ribosomal protein
YGL135W	RPL1B, large subunit ribosomal protein L1
YGL143C	MRF1, mitochondrial peptide chain release factor
YGL165C	Dubious ORF, overlaps with CUP2
YGL206C	CHC1, clathrin heavy chain
YGL218W	Dubious ORF, overlaps with MDM34
YGL237C	HAP2, component of heterotrimeric CCAAT-binding factor
YGL240W	DOC1, anaphase promoting complex (APC10)
YGL244W	RTF1, pol II transcription elongation factor, regulates DNA binding properties of TBP
YGL251C	HFM1, Meiosis specific DNA helicase
YGR020C	VMA7, vacuolar H(+)-ATPase 14 kDa subunit of the catalytic (V0) sector
YGR062C	COX18, required for activity of mitochondrial cytochrome oxidase
YGR076C	MRPL25, mitochondrial ribosomal protein
YGR102C	Unknown function, located in mitochondria
YGR105W	VMA21, required for vacuolar H(+)-ATPase (V-ATPase) assembly
YGR112W	SHY1, mitochondrial protein required for assembly of cytochrome c oxidase complex
YGR150C	Unknown function, located in mitochondria
YGR155W	CYS4, cystathionine beta-synthase
YGR167W	CLC1, clathrin light chain
YGR171C	MSM1, Met-tRNA synthetase, mitochondrial
YGR180C	RNR4, component of ribonucleotide reductase small subunit
YGR215W	RSM27, mitochondrial ribosomal protein
YGR220C	MRPL9, mitochondrial ribosomal protein
YGR222W	PET54, specific translational activator for COX3
YGR243W	FMP43, protein was localized to mitochondria
YGR262C	BUD32, may be involved in polar bud-site selection
YHL038C	CBP2, required for splicing of the COB aI5 intron and 21S mitochondrial rRNA intron
YHR006W	STP2, Transcription factor; activates transcription of amino acid permease genes
YHR009C	Unknown function
YHR011W	DIA4, tRNA synthetase, may be involved in mitochondrial function
YHR026W	PPA1, proteolipid of the vacuolar H(+)-ATPase
YHR038W	RRF1, Mitochondrial ribosome recycling factor, essential for respiratory function
YHR039C	MSC7, Protein of unknown function, GFP-fusion protein in endoplasmic reticulum
YHR039C-B	VMA10, vacuolar H(+)-ATPase (V-ATPase) 13 kDa subunit
YHR049C-A	Dubious ORF
YHR051W	COX6, cytochrome c oxidase subunit VI
YHR060W	VMA22, protein involved in vacuolar H(+)-ATPase assembly or function
YHR067W	HTD2, involved in mitochondrial fatty acid biosynthesis
YHR091C	MSR1, arginyl-tRNA synthetase of mitochondria
YHR120W	MSH1, involved in mitochondrial DNA repair
YHR147C	MRPL6, mitochondrial ribosomal protein
YHR168W	MTG2, mitochondrial GTPase, possibly involved in ribosome assembly
YIL125W	KGD1, alpha-Ketoglutarate dehydrogenase
YIL157C	COA1, required for assembly of the cytochrome c oxidase complex
YIR021W	MRS1, protein involved in mitochondrial RNA splicing of COB mRNA
YJL003W	COX16, required for assembly of cytochrome c oxidase
YJL046W	Similarity to lipoate-protein ligase A
YJL062W-A	Putative protein of unknown function, GFP-fusion protein localizes to the mitochondria
YJL063C	MRPL8, mitochondrial ribosomal protein of the large subunit
YJL096W	MRPL49, mitochondrial ribosomal protein of the large subunit
YJL102W	MEF2, mitochondrial translation elongation factor
YJL120W	Dubious ORF, overlaps with RPE1
YJL121C	RPE1, ribulose-5-phosphate 3-epimerase
YJL124C	LSM1, involved in degradation of cytoplasmic mRNAs
YJL176C	SWI3, component of SWI-SNF global transcription activator complex
YJL180C	ATP12, F1-ATP synthase assembly protein
YJL184W	GON7, unknown function
YJL209W	CBP1, required for COB mRNA stability or 5' processing

YJR040W	GEF1, voltage-gated chloride channel
YJR077C	MIR1, phosphate transporter of the mitochondrial carrier (MCF) family
YJR113C	RSM7, Mitochondrial ribosomal protein of the small subunit
YJR121W	ATP2, beta subunit of F1-ATP synthase
YJR122W	CAF17, component of the CCR4 transcription complex
YJR144W	MGM101, mitochondrial genome maintenance protein
YKL003C	MRP17, mitochondrial ribosomal protein
YKL016C	ATP7, ATP synthase subunit d
YKL040C	NFU1, homeostasis of metal ions, Nifu-like protein (NUB1)
YKL055C	OAR1, mitochondrial type II fatty acid synthase
YKL080W	VMA5, vacuolar H(+)-ATPase (V-ATPase) hydrophilic subunit (subunit C)
YKL109W	HAP4, component of heterotrimeric CCAAT-binding factor
YKL114C	APN1, Major apurinic/apyrimidinic endonuclease, repair of DNA damage
YKL119C	VPH2, vacuolar H(+)-ATPase (V-ATPase) assembly protein acting in the ER
YKL134C	OCT1, mitochondrial intermediate peptidase
YKL138C	MRPL31, mitochondrial ribosomal protein
YKL155C	RSM22, mitochondrial ribosomal protein
YKL169C	Dubious ORF, overlaps with MRPL38
YKL170W	MRPL38, mitochondrial ribosomal protein
YKL194C	MST1, mitochondrial threonyl tRNA synthase
YKR006C	MRPL13, mitochondrial ribosomal protein
YKR085C	MRPL20, mitochondrial ribosomal protein
YLL018C-A	COX19, required for cytochrome c oxidase assembly
YLL027W	ISA1, mitochondrial protein required for normal iron metabolism
YLL033W	Unknown function
YLL041C	SDH2, iron-sulfur protein subunit of succinat dehydrogenase
YLL042C	ATG10, E2-like conjugating enzyme, involved in autophagy
YLR038C	COX12, cytochrome-c oxidase, subunit VIb
YLR056W	ERG3, C-5 sterol desaturase (microsomal membrane)
YLR067C	PET309, required for stability and translation of COX1 mRNA
YLR069C	MEF1, mitochondrial translation elongation factor G
YLR070C	XYL2, Xylitol dehydrogenase, converts xylitol to D-xylulose
YLR091W	Unknown function, located in mitochondria
YLR125W	Unknown function
YLR139C	SLS1, protein involved in mitochondrial metabolism
YLR144C	ACF2, beta-1,3-endoglucanase; probable role in cortical actin cytoskeleton assembly
YLR201C	COQ9, Mitochondrial inner membrane protein required for ubiquinone biosynthesis
YLR202C	Dubious ORF, overlaps with YLR201C and MSS51
YLR203C	MSS51, mitochondrial protein required for respiratory growth and translation of COX1
YLR260W	LCB5, long chain base kinase, involved in sphingolipid metabolism
YLR270W	DCS1, Non-essential hydrolase involved in mRNA decapping
YLR294C	Dubious ORF, overlaps with ATP14
YLR295C	ATP14, ATP synthase subunit h
YLR304C	ACO1, aconitase
YLR312W-A	MRPL15, mitochondrial ribosomal protein
YLR337C	VRP1, involved in cytoskeletal organization and cytokinesis
YLR369W	SSQ1, mitochondrial Hsp70 involved in biogenesis of iron-sulfur proteins
YLR382C	NAM2, leucyl-tRNA synthetase, mitochondrial,
YLR439W	MRPL4, mitochondrial ribosomal protein of the large subunit
YLR447C	VMA6, vacuolar H(+)-ATPase (V-ATPase) 36 kDa subunit
YML061C	PIF1, single-stranded DNA-dependent ATPase and 5'-3' DNA helicase
YML087C	Unknown function
YML110C	COQ5, involved in ubiquinone biosynthesis
YML120C	NDI1, NADH:ubiquinone oxidoreductase
YMR015C	ERG5, C-22 sterol desaturase
YMR021C	MAC1, Copper-sensing transcription factor
YMR035W	IMP2, catalytic subunit of the mitochondrial inner membrane protease Imp
YMR064W	AEP1, required for accumulation of transcript of ATP9/OLI1
YMR070W	MOT3, Nuclear transcription factor; e.g. repression of ergosterol biosynthetic genes

YMR072W	ABF2, DNA-binding protein required for maintenance of mitochondrial genome
YMR077C	VPS20, Myristoylated subunit of the endosomal sorting complex
YMR089C	YTA12, involved in proteolytic and chaperone activities in the inner membrane
YMR097C	MTG1, likely functions in assembly of the large ribosomal subunit
YMR098C	ATP25, required for stability of ATP9 mRNA
YMR150C	IMP1, catalytic subunit of the mitochondrial inner membrane protease Imp
YMR151W	YIM2, Dubious ORF, overlaps with IMP1
YMR158W	MRPS8, Mitochondrial ribosomal protein of the small subunit
YMR188C	MRPS17, Mitochondrial ribosomal protein of the small subunit
YMR193W	MRPL24, mitochondrial ribosomal protein of the large subunit
YMR228W	MTF1, mitochondrial RNA polymerase specificity factor
YMR256C	COX7, cytochrome c oxidase, subunit VII
YMR257C	PET111, required for mitochondrial translation of COX2 mRNA
YMR267W	PPA2, inorganic pyrophosphatase, mitochondrial
YMR282C	AEP2, required for the expression of Atp9p
YMR286W	MRPL33, mitochondrial ribosomal protein of the large subunit
YMR287C	DSS1, RNase, associates with the ribosome, turnover of aberrant RNAs
YMR293C	May be involved in mitochondrial function
YNL005C	MRP7, mitochondrial ribosomal protein
YNL052W	COX5A, cytochrome c oxidase subunit Va
YNL073W	MSK1, lysyl-tRNA synthetase, mitochondrial
YNL081C	Putative mitochondrial ribosomal protein of the small subunit
YNL138W	SRV2, adenylate cyclase-associated protein
YNL159C	ASI2, Integral inner nuclear membrane protein
YNL170W	Dubious ORF, overlaps with PSD1
YNL177C	MRPL22, Mitochondrial ribosomal protein of the large subunit
YNL184C	Dubious ORF, overlaps with MRPL19
YNL185C	MRPL19, Mitochondrial ribosomal protein of the large subunit
YNL213C	Unknown function, located in mitochondria
YNL252C	MRPL17, mitochondrial ribosomal protein
YNL315C	ATP11, F1-ATP synthase assembly protein
YNR020C	ATP23, metalloprotease required for processing of Atp6
YNR037C	RSM19, Mitochondrial ribosomal protein of the small subunit
YNR041C	COQ2, para-hydroxybenzoate-polyprenyltransferase
YNR042W	Dubious ORF, overlaps with COQ2
YNR045W	PET494, translational activator required for mitochondrial translation of COX3
YOL008W	COQ10, Coenzyme Q binding protein
YOL009C	MDM12, mitochondrial morphology and inheritance protein
YOL033W	MSE1, glutamyl-tRNA synthetase, mitochondrial
YOL051W	GAL11, Component of RNA polymerase II holoenzyme
YOL071W	EMI5, non-essential protein of unknown function
YOL083W	Unknown function
YOL095C	HMI1, mitochondrial DNA helicase involved in maintenance of mtDNA
YOL096C	COQ3, catalyzes two different O-methylation steps in ubiquinone biosynthesis
YOR036W	PEP12, syntaxin homolog (t-SNARE) involved in Golgi to vacuole transport
YOR065W	CYT1, cytochrome c1
YOR127W	RGA1, GTPase-activating protein for Cdc42p
YOR150W	MRPL23, Mitochondrial ribosomal protein
YOR155C	ISN1, Inosine 5'-monophosphate (IMP)-specific 5'-nucleotidase, breakdown of IMP
YOR158W	PET123, mitochondrial ribosomal protein
YOR187W	TUF1, translation elongation factor Tu, mitochondrial
YOR200W	Dubious ORF, overlaps with PET56
YOR211C	MGM1, peripheral membrane protein required for mitochondrial morphology
YOR221C	MCT1, mitochondrial type II fatty acid synthase
YOR241W	MET7, required for methionine synthesis and for maintenance of mitochondrial DNA
YOR305W	Unknown function, probably mitochondrial
YOR318C	Dubious ORF unlikely to encode a protein
YOR330C	MIP1, mitochondrial DNA-directed DNA polymerase
YOR331C	Dubious ORF, overlaps with VMA4

YOR332W	VMA4, vacuolar H(+)-ATPase hydrophilic subunit (subunit E)
YOR350C	MNE1, similar to Lucilia illustris mitochondrial cytochrome oxidase
YOR358W	HAP5, component of heterotrimeric CCAAT-binding factor
YOR375C	GDH1, NADP(+)-dependent glutamate dehydrogenase
YOR380W	RDR1, Transcriptional repressor, control of multidrug resistance
YPL013C	MRPS16, Mitochondrial ribosomal protein of the small subunit
YPL031C	PHO85, cyclin-dependent kinase
YPL045W	VPS16, vacuolar sorting protein
YPL059W	GRX5, mitochondrial protein involved in the synthesis/assembly of iron-sulfur centers
YPL078C	ATP4, subunit 4 of F0-ATP synthase
YPL097W	MSY1, tyrosyl-tRNA synthetase, mitochondrial
YPL104W	MSD1, aspartyl-tRNA synthetase, mitochondrial
YPL132W	COX11, required for heme A synthesis
YPL136W	Dubious ORF, overlaps with GIP3
YPL148C	PPT2, activates mitochondrial acyl carrier protein
YPL172C	COX10, farnesyl transferase required for heme A synthesis
YPL173W	MRPL40, mitochondrial ribosomal protein of the large subunit
YPL188W	POS5, mitochondrial NADH kinase; required for the response to oxidative stress
YPL189C-A	COA2, Cytochrome oxidase assembly factor
YPL215W	CBP3, required for assembly of cytochrome bc1 complex
YPL234C	VMA11, proteolipid component of V-ATPase
YPL254W	HFI1, component of the ADA complex
YPL262W	FUM1, fumarate hydratase
YPL271W	ATP15, epsilon subunit of F1-ATP synthase
YPR036W	VMA13, vacuolar H(+)-ATPase (V-ATPase) 54 kDa subunit of V1 sector)
YPR066W	UBA3, Rub1-activating enzyme, similar to ubiquitin-activating E1 protein
YPR067W	ISA2, mitochondrial protein required for iron metabolism
YPR099C	Dubious ORF, overlaps with MRPL51
YPR116W	Unknown function
YPR123C	Dubious ORF, overlaps with CTR1
YPR191W	QCR2, ubiquinol cytochrome c reductase core protein 2
