# Supplementary material for: Genome-wide deletion mutant analysis reveals genes required for respiratory growth, mitochondrial genome maintenance and mitochondrial protein synthesis in Saccharomyces cerevisiae
Source: Genome Biol. 2009 Sep 14;10(9):R95. doi: 10.1186/gb-2009-10-9-r95 (PMC2768984; doi:10.1186/gb-2009-10-9-r95)
Supplement: Additional data file 2 — pet genes unique to this study. [file gb-2009-10-9-r95-S2.PDF]

Supplemental table 2. *pet* genes unique to this study. Genes were grouped according to the function of the encoded gene products by the same criteria used in Supplemental table 3 (see Additional data file 3).

KNOWN MITOCHONDRIAL PROTEINS

Mitochondrial protein synthesis
<u>Mitochondrial ribosomal subunits</u>

YBR146W	MRPS9, Mitochondrial ribosomal protein of the small subunit
YDR116C	MRPL1, Mitochondrial ribosomal protein of the large subunit
YNL185C	MRPL19, Mitochondrial ribosomal protein of the large subunit

Respiratory chain
<u>F0/F1 ATP synthase (complex V)</u>

YBR039W	ATP3, Gamma subunit of the F1 sector of mitochondrial F1F0 ATP synthase

Mitochondrial proteases and peptidases

YEL059C-A	SOM1, Subunit of the mitochondrial inner membrane peptidase, maturation of proteins

Other mitochondrial factors

YPL188W	POS5, mitochondrial NADH kinase; required for the response to oxidative stress

KNOWN NON-MITOCHONDRIAL PROTEINS

Vacuole
<u>Vacuolar inheritance and protein sorting factors</u>

YDL077C	VAM6, Vacuolar protein, tethers steps of vacuolar membrane fusion
YBR097W	VPS15, serine/threonine protein kinase involved in vacuolar protein sorting

Lipids biosynthesis, non-mitochondrial or unspecified

YMR015C	ERG5, C-22 sterol desaturase

Other

YAL013W	DEP1, transcriptional modulator
YAL026C	DRS2, maintains membrane lipid asymmetry in post-Golgi secretory vessicles
YBL019W	APN2, Class II abasic (AP) endonuclease; repair of DNA damage; homolog of hHAP1
YBL031W	SHE1 Cytoskeletal protein of unknown function; overexpression causes growth arrest
YBL032W	HEK2, RNA binding protein; localizes ASH1 mRNA
YBL036C	non-specific single-domain racemase
YBL046W	PSY4, regulatory subunit of a protein phosphatase complex (nuclear)
YBL057C	PTH2, negatively regulates the ubiquitin-proteasome pathway
YBL082C	ALG3, alpha(1-3) mannosyltransferase
YBL093C	ROX3, RNA polymerase II holoenzyme component
YBR128C	ATG14, Subunit of an autophagy-specific phosphatidylinositol 3-kinase complex
YBR283C	SSH1, involved in co-translational protein translocation in the ER
YCL010C	SGF29, Probable 29kKDa Subunit of SAGA histone acetyltransferase complex
YCR020W-B	HTL1, Subunit of the RSC chromatin remodeling complex
YDL033C	SLM3, tRNA-specific 2-thiouridylase
YDL039C	PRM7, Pheromone-regulated protein
YDL091C	UBX3, UBX domain-containing protein that interacts with Cdc48p
YDL192W	ARF1, GTPase of the Ras superfamily involved in coated vesicles formation
YDR025W	RPS11A, Protein component of the small (40S) ribosomal subunit
YDR349C	YPS7, Putative GPI-anchored aspartic protease
YDR448W	ADA2, component of the histone acetyltransferase complexes
YDR523C	SPS1, Putative protein serine/threonine kinase
YER114C	BOI2, Protein implicated in polar growth, functionally redundant with Boi1p
YER131W	RPS26B, Protein component of the small (40S) ribosomal subunit
YER155C	BEM2, Rho GTPase activating protein; control of cytoskeleton organization

YGL017W	ATE1, Arginyl-tRNA-protein transferase
YGL251C	HFM1, Meiosis specific DNA helicase
YGR262C	BUD32, may be involved in polar bud-site selection
YHR006W	STP2, Transcription factor; activates transcription of amino acid permease genes
YJL124C	LSM1, involved in degradation of cytoplasmic mRNAs
YKL114C	APN1, Major apurinic/apyrimidinic endonuclease, repair of DNA damage
YLL042C	ATG10, E2-like conjugating enzyme, involved in autophagy
YLR070C	XYL2, Xylitol dehydrogenase, converts xylitol to D-xylulose
YLR144C	ACF2, beta-1,3-endoglucanase; probable role in cortical actin cytoskeleton assembly
YMR070W	MOT3, Nuclear transcription factor; e.g. repression of ergosterol biosynthetic genes
YMR077C	VPS20, Myristoylated subunit of the endosomal sorting complex
YNL159C	ASI2, Integral inner nuclear membrane protein
YOL051W	GAL11, Component of RNA polymerase II holoenzyme
YOR127W	RGA1, GTPase-activating protein for Cdc42p
YOR155C	ISN1, Inosine 5'-monophosphate (IMP)-specific 5'-nucleotidase, breakdown of IMP
YOR375C	GDH1, NADP(+)-dependent glutamate dehydrogenase
YOR380W	RDR1, Transcriptional repressor, control of multidrug resistance
YPR066W	UBA3, Rub1-activating enzyme, similar to ubiquitin-activating E1 protein

UNKNOWN PROTEINS

Unknown function

YCL001W-A	Unknown function
YDL012C	Plasma membrane protein of unknown function
YDL099W	BUG1, unknown function; GFP-fusion protein in the cytoplasm in a punctate pattern
YDL114W	Putative protein of unknown function with similarity to acyl-carrier-protein reductases
YDL129W	Unknown function
YDL133W	Unknown function
YDL157C	unknown function; detected in highly purified mitochondria in high-throughput studies
YDR458C	HEH2, Protein of unknown function; GFP-fusion protein in nuclear periphery
YGR243W	FMP43, protein was localized to mitochondria
YHR009C	Unknown function
YHR039C	MSC7, Protein of unknown function, GFP-fusion protein in endoplasmic reticulum
YIL157C	FMP35, Unknown function
YJL062W-A	Putative protein of unknown function, GFP-fusion protein localizes to the mitochondria
YJL184W	GON7, unknown function
YLR125W	Unknown function
YML087C	Unknown function
YNR020C	Unknown function
YOL083W	Unknown function
YPL189C-A	Unknown function

Questionable ORFs

YBL053W	Dubious ORF, overlaps with SAS3n
YBL062W	Dubious ORF, overlaps with SKT5
YDR010C	Dubious ORF
YDR269C	Dubious ORF, overlaps with CCC2
YDR271C	Dubious ORF, overlaps with CCC2
YDR491C	Dubious ORF, overlaps with IZH1
YGL165C	Dubious ORF, overlaps with CUP2
YHR049C-A	Dubious ORF
YLR294C	Dubious ORF, overlaps with ATP14
YOR318C	Dubious ORF unlikely to encode a protein
YPL136W	Dubious ORF, overlaps with GIP3
