# Supplementary material for: Genome-wide deletion mutant analysis reveals genes required for respiratory growth, mitochondrial genome maintenance and mitochondrial protein synthesis in Saccharomyces cerevisiae
Source: Genome Biol. 2009 Sep 14;10(9):R95. doi: 10.1186/gb-2009-10-9-r95 (PMC2768984; doi:10.1186/gb-2009-10-9-r95)
Supplement: Additional data file 3 — pet genes grouped according to their occurrence in pet screens and localization and function of the encoded gene products. [file gb-2009-10-9-r95-S3.PDF]

Supplemental table 3. *pet* genes grouped according to their occurrence in *pet* screens and localization and function of the encoded gene products. The list indicates systematic and standard names and a brief description of gene function according to the Saccharomyces Genome Database and manual annotation.

176 *pet* genes found in three screens (163 genes excluding questionable ORFs)

KNOWN MITOCHONDRIAL PROTEINS (129 GENES)

Mitochondrial DNA metabolism (8 genes)

YCR028C-A	RIM1, binds single-stranded DNA, required for DNA replication in mitochondria
YDR296W	MHR1, involved in repair, recombination and maintenance of mitochondrial DNA
YHR120W	MSH1, involved in mitochondrial DNA repair
YJR144W	MGM101, mitochondrial genome maintenance protein
YML061C	PIF1, single-stranded DNA-dependent ATPase and 5'-3' DNA helicase
YMR072W	ABF2, DNA-binding protein required for maintenance of mitochondrial genome
YOL095C	HMI1, mitochondrial DNA helicase involved in maintenance of mitochondrial genome
YOR330C	MIP1, mitochondrial DNA-directed DNA polymerase

Mitochondrial RNA synthesis (7 genes)

YDL044C	MTF2, mitochondrial protein involved in mRNA splicing and protein synthesis
YDR194C	MSS116, mitochondrial RNA helicase, required for splicing of group II introns
YHL038C	CBP2, required for splicing of the COB aI5 intron and 21S mitochondrial rRNA intron
YIR021W	MRS1, protein involved in mitochondrial RNA splicing of COB mRNA
YLR067C	PET309, required for stability and translation of COX1 mRNA
YMR098C	ATP25, required for stability of ATP9 mRNA
YMR228W	MTF1, mitochondrial RNA polymerase specificity factor

Mitochondrial protein synthesis (63 genes)

<u>Mitochondrial ribosomal subunits (39 genes)</u>

YBL090W	MRP21, mitochondrial ribosomal protein
YBR282W	MRPL27, mitochondrial ribosomal protein
YCR003W	MRPL32, mitochondrial ribosomal protein
YCR046C	IMG1, mitochondrial ribosomal protein
YCR071C	IMG2, mitochondrial ribosomal protein
YDL045W-A	MRP10, mitochondrial ribosomal protein
YDR115W	Putative mitochondrial ribosomal protein
YDR175C	RSM24, mitochondrial ribosomal protein
YDR237W	MRPL7, mitochondrial ribosomal protein
YDR337W	MRPS28, mitochondrial ribosomal protein
YER050C	RSM18, mitochondrial ribosomal protein
YGL129C	RSM23, mitochondrial ribosomal protein
YGR076C	MRPL25, mitochondrial ribosomal protein
YGR215W	RSM27, mitochondrial ribosomal protein
YGR220C	MRPL9, mitochondrial ribosomal protein
YHR147C	MRPL6, mitochondrial ribosomal protein
YJL063C	MRPL8, mitochondrial ribosomal protein
YJL096W	MRPL49, mitochondrial ribosomal protein
YJR113C	RSM7, mitochondrial ribosomal protein
YKL003C	MRP17, mitochondrial ribosomal protein
YKL138C	MRPL31, mitochondrial ribosomal protein
YKL155C	RSM22, mitochondrial ribosomal protein
YKL170W	MRPL38, mitochondrial ribosomal protein
YKR006C	MRPL13, mitochondrial ribosomal protein
YKR085C	MRPL20, mitochondrial ribosomal protein
YLR312W-A	MRPL15, mitochondrial ribosomal protein
YLR439W	MRPL4, mitochondrial ribosomal protein
YMR158W	MRPS8, mitochondrial ribosomal protein

YMR193W	MRPL24, mitochondrial ribosomal protein
YMR286W	MRPL33, mitochondrial ribosomal protein
YNL005C	MRP7, mitochondrial ribosomal protein
YNL081C	Putative mitochondrial ribosomal protein of the small subunit
YNL177C	MRPL22, mitochondrial ribosomal protein
YNL252C	MRPL17, mitochondrial ribosomal protein
YNR037C	RSM19, mitochondrial ribosomal protein
YOR150W	MRPL23, mitochondrial ribosomal protein
YOR158W	PET123, mitochondrial ribosomal protein
YPL013C	MRPS16, mitochondrial ribosomal protein
YPL173W	MRPL40, mitochondrial ribosomal protein
Mitochondrial tRNA synthetases (10 genes)	
YCR024C	SLM5, Asparaginyl-tRNA synthetase, mitochondrial
YER087W	Similarity to tRNA synthetases; protein is detected in mitochondria
YGR171C	MSM1, Met-tRNA synthetase, mitochondrial
YHR011W	DIA4, tRNA synthetase, may be involved in mitochondrial function
YHR091C	MSR1, arginyl-tRNA synthetase, mitochondrial
YLR382C	NAM2, leucyl-tRNA synthetase, mitochondrial
YNL073W	MSK1, lysyl-tRNA synthetase, mitochondrial
YOL033W	MSE1, glutamyl-tRNA synthetase, mitochondrial
YPL097W	MSY1, tyrosyl-tRNA synthetase, mitochondrial
YPL104W	MSD1, aspartyl-tRNA synthetase, mitochondrial
Other (14 genes)	
YDR197W	CBS2, translational activator for cyt b
YGL143C	MRF1, mitochondrial peptide chain release factor
YGR222W	PET54, specific translational activator for COX3
YHR038W	RRF1, mitochondrial ribosome recycling factor, essential for respiratory function
YHR168W	MTG2, mitochondrial GTPase, possibly involved in ribosome assembly
YJL102W	MEF2, mitochondrial translation elongation factor
YLR069C	MEF1, mitochondrial translation elongation factor G
YLR203C	MSS51, mitochondrial protein required for respiratory growth and translation of COX1
YMR064W	AEP1, required for accumulation of transcript of ATP9/OLI1
YMR097C	MTG1, likely functions in assembly of the large ribosomal subunit
YMR257C	PET111, required for mitochondrial translation of COX2 mRNA
YMR282C	AEP2, required for the expression of Atp9p
YMR287C	DSS1, RNase, associates with the ribosome, turnover of aberrant RNAs
YOR187W	TUF1, translation elongation factor Tu, mitochondrial

Respiratory chain (24 genes)

Cytochrome bc1 complex (Ubiquinol-cytochrome c reductase complex, complex III) (4 genes)	
YBL045C	COR1, ubiquinol cytochrome c reductase core protein 1
YEL024W	RIP1, ubiquinol cytochrome c reductase iron-sulfur protein
YOR065W	CYT1, cytochrome c1
YPR191W	QCR2, ubiquinol cytochrome c reductase core protein 2
Cytochrome c oxidase (complex IV) (5 genes)	
YDL067C	COX9, cytochrome c oxidase subunit VIIA
YHR051W	COX6, cytochrome c oxidase subunit VI
YLR038C	COX12, cytochrome-c oxidase, subunit VIb
YMR256C	COX7, cytochrome c oxidase, subunit VII
YNL052W	COX5A, cytochrome c oxidase subunit Va
F0/F1 ATP synthase (complex V) (7 genes)	
YBL099W	ATP1, alpha subunit of F1-ATP synthase
YDR298C	ATP5, subunit 5 of F0-ATP synthase, oligomycin sensitivity-conferring subunit
YDR377W	ATP17, ATP synthase subunit f
YJR121W	ATP2, beta subunit of F1-ATP synthase
YKL016C	ATP7, ATP synthase subunit d
YPL078C	ATP4, subunit 4 of F0-ATP synthase
YPL271W	ATP15, epsilon subunit of F1-ATP synthase
Assembly factors (8 genes)	
YAL039C	CYC3, holocytochrome c synthase (cytochrome c heme lyase)
YDR079W	PET100, required for assembly of cytochrome c oxidase
YGR062C	COX18, required for activity of mitochondrial cytochrome oxidase

YGR112W	SHY1, mitochondrial protein required for assembly of cytochrome c oxidase complex
YJL180C	ATP12, F1-ATP synthase assembly protein
YLL018C-A	COX19, required for cytochrome c oxidase assembly
YNL315C	ATP11, F1-ATP synthase assembly protein
YPL215W	CBP3, required for assembly of cytochrome bc1 complex

Mitochondrial enzymes (11 genes)

YAL044C	GCV3, glycine decarboxylase hydrogen carrier protein H subunit
YBR003W	COQ1, hexaprenyl pyrophosphate synthetase
YDR148C	KGD2, 2-oxoglutarate dehydrogenase complex E2 component
YDR204W	COQ4, involved in biosynthesis of coenzyme Q
YER061C	CEM1, beta-ketoacyl-ACP synthase, mitochondrial
YLR201C	COQ9, mitochondrial inner membrane protein required for ubiquinone biosynthesis
YLR304C	ACO1, aconitase
YMR267W	PPA2, inorganic pyrophosphatase, mitochondrial
YNR041C	COQ2, para-hydroxybenzoate-polyprenyltransferase
YPL132W	COX11, required for heme A synthesis
YPL172C	COX10, farnesyl transferase required for heme A synthesis

Lipids biosynthesis, mitochondrial (5 genes)

YBR026C	ETR1, localized to in mitochondria, where it has a probable role in fatty acid synthesis
YHR067W	HTD2, involved in mitochondrial fatty acid biosynthesis
YKL055C	OAR1, mitochondrial type II fatty acid synthase
YOR221C	MCT1, mitochondrial type II fatty acid synthase
YPL148C	PPT2, activates mitochondrial acyl carrier protein

Mitochondrial proteases and peptidases (4 genes)

YER017C	AFG3, involved in proteolytic and chaperone activities at the inner membrane
YKL134C	OCT1, mitochondrial intermediate peptidase
YMR089C	YTA12, involved in proteolytic and chaperone activities in the inner membrane
YMR150C	IMP1, catalytic subunit of the mitochondrial inner membrane protease Imp

Mitochondrial morphology (1 gene)

YOR211C	MGM1, peripheral membrane protein required for mitochondrial morphology

Other mitochondrial factors (6 genes)

YDL107W	MSS2, required for export of C-terminal tail of Cox2p through the inner membrane
YER154W	OXA1, component of the mitochondrial protein export machinery
YFL016C	MDJ1, DnaJ co-chaperone involved in mitochondrial biogenesis and protein folding
YLL027W	ISA1, mitochondrial protein required for normal iron metabolism
YLR139C	SLS1, protein involved in mitochondrial metabolism
YPR067W	ISA2, mitochondrial protein required for iron metabolism

KNOWN NON-MITOCHONDRIAL PROTEINS (26 GENES)

Vacuole (12 genes)

<u>V-ATPase subunits (9 genes)</u>

YBR127C	VMA2, V-ATPase regulatory subunit
YDL185W	VMA1, V-ATPase catalytic subunit (subunit A)
YEL027W	CUP5, V-ATPase 16 kDa proteolipid subunit of membrane (V0) sector
YEL051W	VMA8, V-ATPase subunit of the V1 catalytic sector
YHR039C-B	VMA10, V-ATPase 13 kDa subunit
YKL080W	VMA5, V-ATPase hydrophilic subunit (subunit C)
YLR447C	VMA6, V-ATPase 36 kDa subunit
YOR332W	VMA4, V-ATPase hydrophilic subunit (subunit E)
YPL234C	VMA11, V-ATPase proteolipid component

<u>V-ATPase assembly (2 genes)</u>

YGR105W	VMA21, required for V-ATPase assembly
YHR060W	VMA22, involved in V-ATPase assembly or function

<u>Vacuolar inheritance and protein sorting factors (1 gene)</u>

YPL045W	VPS16, vacuolar sorting protein

Lipids biosynthesis, non-mitochondrial or unspecified (1 gene)
YLR260W LCB5, long chain base kinase, involved in sphingolipid metabolism

Transcription (nuclear) (8 genes)
Heterotrimeric CCAAT-binding factor (4 genes)
YBL021C HAP3, component of heterotrimeric CCAAT-binding factor
YGL237C HAP2, component of heterotrimeric CCAAT-binding factor
YKL109W HAP4, component of heterotrimeric CCAAT-binding factor
YOR358W HAP5, component of heterotrimeric CCAAT-binding factor
Other (4 genes)
YGL070C RPB9, RNA polymerase II, non-essential subunit, not shared
YGL071W RCS1, transcription factor regulates genes involved in iron uptake and cell size
YJL176C SWI3, component of SWI-SNF global transcription activator complex
YJR122W CAF17, component of the CCR4 transcription complex

Non-mitochondrial metabolic enzymes (1 gene)
YOR241W MET7, required for methionine synthesis and for maintenance of mitochondrial DNA

Other (4 genes)
YER014C-A BUD25, involved in bipolar budding
YER145C FTR1, iron permease that mediates high-affinity iron uptake
YGL135W RPL1B, large subunit ribosomal protein L1
YGR180C RNR4, component of ribonucleotide reductase small subunit

UNKNOWN PROTEINS (21 GENES)

Unknown function (8 genes)
YDR065W RRG1, unknown function
YGR150C RRG2, unknown function, located in mitochondria
YJL046W RRG3, unknown function, similarity to lipoate-protein ligase A
YLL033W RRG4, unknown function
YLR091W RRG5, unknown function, located in mitochondria
YMR293C RRG6 (HER2), may be involved in mitochondrial function
YOR305W RRG7, unknown function, probably mitochondrial
YPR116W RRG8, unknown function

Questionable ORFs (13 genes)
YBL100C Dubious ORF, overlaps with ATP1
YCL007C Dubious ORF; overlaps verified ORF YCL005W-A
YDL068W Dubious ORF, overlaps with CBS1
YDR114C Dubious ORF, overlaps with YDR115W
YDR230W Dubious ORF, overlaps with COX20
YGL218W Dubious ORF, overlaps with MDM34
YJL120W Dubious ORF, overlaps with RPE1
YKL169C Dubious ORF, overlaps with MRPL38
YLR202C Dubious ORF, overlaps with YLR201C and MSS51
YNL184C Dubious ORF, overlaps with MRPL19
YOR200W Dubious ORF, overlaps with PET56
YOR331C Dubious ORF, overlaps with VMA4
YPR099C Dubious ORF, overlaps with MRPL51

120 *pet* genes found in two of three screens (114 genes excluding questionable ORFs)

KNOWN MITOCHONDRIAL PROTEINS (63 GENES)

Mitochondrial RNA synthesis (3 genes)

YFL036w	RPO41, mitochondrial RNA polymerase
YJL209W	CBP1, required for COB mRNA stability or 5' processing
YPL029W	SUV3, mitochondrial RNA helicase

Mitochondrial protein synthesis (20 genes)

Mitochondrial ribosomal subunits (13 genes)

YBL038W	MRPL16, mitochondrial ribosomal protein
YBR251W	MRPS5, mitochondrial ribosomal protein
YBR268W	MRPL37, mitochondrial ribosomal protein
YDL202W	MRPL11, mitochondrial ribosomal protein
YDR347W	MRP1, mitochondrial ribosomal protein
YEL050C	RML2, mitochondrial ribosomal protein
YMR188C	MRPS17, mitochondrial ribosomal protein
YNL284C	MRPL10, mitochondrial ribosomal protein
YNR036C	Mitochondrial protein, similar to human mitochondrial S12 ribosomal proteins
YPL118W	MRP51, mitochondrial ribosomal protein
YPL183W-A	Possible mitochondrial ribosomal protein
YPR100W	MRPL51, mitochondrial ribosomal protein
YPR166C	MRP2, mitochondrial ribosomal protein

Mitochondrial tRNA synthetases (2 genes)

YDR268W	MSW1, Trp-tRNA synthetase, mitochondrial
YPR047W	MSF1, Phe-tRNA synthetase, mitochondrial

Other (5 genes)

YBL080C	PET112, required for mitochondrial translation
YDL069C	CBS1, translational activator of COB mRNA
YER153C	PET122, translational activator required for mitochondrial translation of COX3
YNR045W	PET494, translational activator required for mitochondrial translation of COX3
YOL023W	IFM1, mitochondrial translation initiation factor 2

Respiratory chain (18 genes)

Succinate dehydrogenase complex (complex II) (2 genes)

YKL148C	SDH1, succinate dehydrogenase (ubiquinone) flavoprotein (Fp) subunit
YLL041C	SDH2, iron-sulfur protein subunit of succinat dehydrogenase

Cytochrome bc1 complex (Ubiquinol-cytochrome c reductase complex, complex III) (2 genes)

YDR529C	QCR7, ubiquinol cytochrome c reductase subunit 7
YJL166W	QCR8, Subunit 8 of ubiquinol cytochrome-c reductase complex

F0/F1 ATP synthase (complex V) (2 genes)

YLR295C	ATP14, ATP synthase subunit h
YML081C-A	ATP18, ATP synthase subunit i (or subunit j)

Assembly factors (12 genes)

YBR037C	SCO1, role in copper transport or insertion of copper into cytochrome oxidase
YDR231C	COX20, involved in maturation of Cox2p and its assembly into COX
YDR350C	ATP22, required for assembly of the F0 sector of mitochondrial F1F0 ATP synthase
YDR375C	BCS1, required for expression of functional Rieske iron-sulfur protein
YER058W	PET117, involved in assembly of cytochrome oxidase
YER141W	COX15, required for cytochrome oxidase assembly
YGR174C	CBP4, ubiquinol-cytochrome c reductase assembly factor
YJL003W	COX16, required for assembly of cytochrome c oxidase
YKL087C	CYT2, holocytochrome-c1 synthase (CC1HL)
YLL009C	COX17, copper metallochaperone for cytochrome c oxidase
YLR393W	ATP10, required for F1-F0 ATP synthase assembly
YML129C	COX14, required for assembly of cytochrome oxidase

Mitochondrial enzymes (8 genes)

YFL018C	LPD1, dihydrolipoamide dehydrogenase, pyruvate dehydrogenase complex

YGR255C COQ6, monooxygenase required for coenzyme Q (ubiquinone) biosynthesis
YIL125W KGD1, alpha-Ketoglutarate dehydrogenase
YML110C COQ5, involved in ubiquinone biosynthesis
YML120C NDI1, NADH:ubiquinone oxidoreductase
YOL096C COQ3, catalyzes two different O-methylation steps in ubiquinone biosynthesis
YOR125C CAT5, mitochondrial inner membrane protein involved in ubiquinone biosynthesis
YPL262W FUM1, fumarate hydratase

Lipids biosynthesis, mitochondrial (1 gene)
YOR196C LIP5, lipoic acid synthase (mitochondrial matrix)

Mitochondrial proteases and peptidases (1 gene)
YMR035W IMP2, catalytic subunit of the mitochondrial inner membrane protease Imp

Mitochondrial morphology (3 genes)
YAL010C MDM10, involved in mitochondrial morphology and inheritance
YBR179C FZO1, transmembrane GTPase required for mitochondrial fusion
YOL009C MDM12, mitochondrial morphology and inheritance protein

Other mitochondrial factors (9 genes)
YDL198C YHM1, protein of the mitochondrial carrier (MCF) family
YGL107C RMD9, mitochondrial protein required for sporulation
YJR077C MIR1, phosphate transporter of the mitochondrial carrier (MCF) family
YLR239C LIP2, lipoyl ligase, involved in the modification of mitochondrial enzymes
YLR369W SSQ1, mitochondrial Hsp70 involved in biogenesis of iron-sulfur proteins
YNL003C PET8, mitochondrial carrier (MCF) family
YOL008W COQ10, coenzyme Q binding protein
YPL005W AEP3, stabilizes the bicistronic AAP1-ATP6 mRNA
YPL059W GRX5, mitochondrial protein involved in the synthesis/assembly of iron-sulfur centers

KNOWN NON-MITOCHONDRIAL PROTEINS (44 GENES)

Vacuole (11 genes)
<u>V-ATPase subunits (3 genes)</u>
YGR020C VMA7, V-ATPase 14 kDa subunit of the catalytic (V0) sector
YHR026W PPA1, proteolipid of the V-ATPase
YPR036W VMA13, V-ATPase 54 kDa subunit of V1 sector
<u>V-ATPase assembly (1 gene)</u>
YKL119C VPH2, V-ATPase assembly protein acting in the ER
<u>Vacuolar inheritance and protein sorting factors (7 genes)</u>
YDR323C PEP7, vacuolar segregation protein required for vacuole inheritance
YKL002W DID4, class E vacuolar protein-sorting (vps) factor
YKL054C VID31, involved in vacuolar import and degradation
YLR148W PEP3, vacuolar protein involved in vacuolar protein sorting
YLR240W VPS34, phosphatidylinositol 3-kinase required for vacuolar protein sorting
YLR396C VPS33, vacuolar sorting protein of the Sec1p family
YOR036W PEP12, syntaxin homolog (t-SNARE) involved in Golgi to vacuole transport

Transcription (nuclear) (5 genes)
YBR289W SNF5, component of SWI-SNF global transcription activator complex
YDL056W MBP1, transcription factor that collaborates with Swi6p
YGL115W SNF4, involved in derepression of glucose-repressed genes
YMR021C MAC1, copper-sensing transcription factor
YPL254W HFI1, component of the ADA complex

Non-mitochondrial metabolic enzymes (4 genes)
YAL012W CYS3, cystathionine gamma-lyase
YGR155W CYS4, cystathionine beta-synthase
YJL101C GSH1, gamma-glutamylcysteine synthetase
YLR377C FBP1, fructose-1,6-bisphosphatase

Other (24 genes)

YAL009W	SPO7, ER membrane protein of unknown function
YAL016W	TPD3, protein serine/threonine phosphatase 2A (PP2A) regulatory subunit A
YAL047C	SPC72, component of the cytoplasmic plaque of the spindle pole body
YDR195W	REF2, involved in mRNA 3'-end formation prior to polyadenylation
YDR270W	CCC2, copper-transporting P-type ATPase
YDR364C	CDC40, pre-mRNA splicing factor
YDR477W	SNF1, serine/threonine protein kinase, for derepression of glucose-repressed genes
YER070W	RNR1, ribonucleosid-diphosphate-reductase, large (R1) subunit
YGL206C	CHC1, clathrin heavy chain
YGL240W	DOC1, anaphase promoting complex (APC10)
YGL244W	RTF1, pol II transcription elongation factor, regulates DNA binding properties of TBP
YGR167W	CLC1, clathrin light chain
YIL018W	RPL2B, ribosomal protein L2
YIL036W	CST6, similarity to Mei4p and to cAMP response element binding proteins
YJL121C	RPE1, ribulose-5-phosphate 3-epimerase
YJR040W	GEF1, voltage-gated chloride channel
YJR090C	GRR1, F-box protein required for glucose repression
YKL040C	NFU1, homeostasis of metal ions, Nifu-like protein (NUB1)
YLR270W	DCS1, non-essential hydrolase involved in mRNA decapping
YLR337C	VRP1, involved in cytoskeletal organization and cytokinesis
YMR058W	FET3, cell surface ferroxidase
YNL138W	SRV2, adenylate cyclase-associated protein
YPL031C	PHO85, cyclin-dependent kinase
YPR124W	CTR1, copper transport protein

UNKNOWN PROTEINS (13 GENES)

Unknown function (7 genes)

YBR163W	Unknown function
YDR332W	Unknown function
YGR102C	Unknown function, located in mitochondria
YMR066W	Unknown function, located in mitochondria
YOL071W	Unknown function
YOR205C	Unknown function, located in mitochondria
YOR350C	Unknown function, similar to Lucilia illustris mitochondrial cytochrome oxidase

Questionable ORFs (6 genes)

YGR219W	Dubious ORF, overlaps with MRPL9
YKL118W	Dubious ORF, overlaps with VPH2
YMR151W	Dubious ORF, overlaps with IMP1
YNL170W	Dubious ORF, overlaps with PSD1
YNR042W	Dubious ORF, overlaps with COQ2
YPR123C	Dubious ORF, overlaps with CTR1

224 *pet* genes found in one of three screens (202 genes excluding questionable ORFs)

KNOWN MITOCHONDRIAL PROTEINS (36 GENES)

Mitochondrial RNA synthesis (3 genes)
YGL064C MRH4, mitochondrial RNA helicase
YKL208W CBT1, required for 3' end processing of the mitochondrial COB mRNA
YPR134W MSS18, involved in splicing a15beta intron of the mitochondrial COX1 transcript

Mitochondrial protein synthesis (11 genes)
Mitochondrial ribosomal subunits (8 genes)
YBR146W MRPS9, mitochondrial ribosomal protein
YDR116C MRPL1, mitochondrial ribosomal protein
YDR322W MRPL35, mitochondrial ribosomal subunit
YDR462W MRPL28, mitochondrial ribosomal protein
YGR165W MRPS35, mitochondrial ribosomal protein
YIL093C RSM53, mitochondrial ribosome small subunit
YKL167C MRP49, mitochondrial ribosomal protein
YMR024W MRPL3, mitochondrial ribosomal protein
Mitochondrial tRNA synthetases (1 gene)
YPL040C ISM1, isoleucyl-tRNA synthetase, mitochondrial
Other (2 genes)
YBR120C CBP6, required for translation of the mitochondrial COB mRNA
YOR201C PET56, ribose methyltransferase for mitochondrial 21S rRNA

Respiratory chain (6 genes)
Cytochrome bc1 complex (Ubiquinol-cytochrome c reductase complex, complex III) (1 gene)
YGR183C QCR9, ubiquinol cytochrome c reductase subunit 9
Assembly factors (5 genes)
YIL157C COA1, required for assembly of the cytochrome c oxidase complex
YJR034W PET191, involved in assembly of cytochrome oxidase
YKL137W CMC1, conserved copper binding protein of the mitochondrial inner membrane
YNR020C ATP23, metalloprotease required for processing of Atp6
YOR037W CYC2, mitochondrial protein, likely participates in ligation of heme to cytochrome c

Mitochondrial enzymes (3 genes)
YDR226W ADK1, adenylate kinase, cytoplasmic and mitochondrial
YHR008C SOD2, manganese superoxide dismutase, mitochondrial
YMR083W ADH3, mitochondrial alcohol dehydrogenase isozyme III

Lipids biosynthesis, mitochondrial (1 gene)
YMR207C HFA1, mitochondrial acetyl-coenzyme A carboxylase, fatty acid biosynthesis

Mitochondrial proteases and peptidases (2 genes)
YBL022C PIM1, mitochondrial ATP-dependent protease
YGR101W PCP1, mitochondrial serine protease, processing of various mitochondrial proteins

Mitochondrial morphology (5 genes)
YAL048C GEM1, GTPase which regulates mitochondrial morphology
YHR194W MDM31, inner membrane protein required for normal mitochondrial morphology
YLL006W MMM1, essential for maintenance of mitochondrial shape and structure
YOL027C MDM38, mitochondrial Distribution and Morphology
YOR147W MDM32, inner membrane protein required for normal mitochondrial morphology

Other mitochondrial factors (5 genes)
YGR257C MTM1, mitochondrial carrier family (MCF) of membrane transporters
YLR204W QRI5, mitochondrial protein of unknown function
YMR060C TOM37, mitochondrial outer membrane sorting and assembly machinery complex
YPL060W LPE10, mitochondrial inner membrane magnesium transporter
YPL188W POS5, mitochondrial NADH kinase; required for the response to oxidative stress

KNOWN NON-MITOCHONDRIAL PROTEINS (123 GENES)

Vacuole (7 genes)

Vacuolar inheritance and protein sorting factors (7 genes)

YDL077C	VAM6, Vacuolar protein, tethers steps of vacuolar membrane fusion
YDR027C	LUV1, Vps52p-Vps53p-Vps54p complex, involved in protein sorting in the Golgi
YDR495C	VPS3, required for the sorting and processing of soluble vacuolar proteins
YJL029C	VPS53, subunit of the Vps52p-Vps53p-Vps54p complex
YGL095C	VPS45, Sec1p family of vacuolar protein sorting
YKR001C	VPS1, vacuolar sorting protein, member of the dynamin family of GTPases
YLL040C	VPS13, involved in vacuolar sorting

Lipids biosynthesis, non-mitochondrial or unspecified (2 genes)

YLR056W	ERG3, C-5 sterol desaturase (microsomal membrane)
YMR015C	ERG5, C-22 sterol desaturase

Transcription (nuclear) (7 genes)

YBR112C	SSN6, general repressor of RNA polymerase II transcription
YCR084C	TUP1, repressor of RNA polymerase II transcription
YER068W	MOT2 (SIG1), zinc finger transcriptional repressor
YHL025W	SNF6, component of SWI-SNF global transcription activator complex
YIL154C	IMP2, transcriptional activator involved in maintenance of ion homeostasis
YMR280C	CAT8, transcription factor required for derepression of gluconeogenic enzymes
YOR290C	SNF2, component of SWI-SNF global transcription activator complex

Non-mitochondrial metabolic enzymes (1 gene)

YNL117W	MLS1, malate synthase, enzyme of the glyoxylate cycle

Other (108 genes)

YAL013w	DEP1, transcriptional modulator
YAL026C	DRS2, maintains membrane lipid asymmetry in post-Golgi secretory vessicles
YBL002W	HTB2, histone H2B subtype
YBL007C	SLA1, involved in assembly of cortical actin cytoskeleton
YBL019W	APN2, class II abasic (AP) endonuclease; repair of DNA damage; homolog of hHAP1
YBL031W	SHE1 cytoskeletal protein of unknown function; overexpression causes growth arrest
YBL032W	HEK2, RNA binding protein; localizes ASH1 mRNA
YBL036C	non-specific single-domain racemase
YBL046W	PSY4, regulatory subunit of a protein phosphatase complex (nuclear)
YBL057C	PTH2, negatively regulates the ubiquitin-proteasome pathway
YBL082C	ALG3, alpha(1-3) mannosyltransferase
YBL093C	ROX3, RNA polymerase II holoenzyme component
YBR035C	PDX3, pyridoxine (pyridoxamine) phosphate oxidase
YBR036C	CSG2, endoplasmic reticulum membrane protein
YBR128C	ATG14, subunit of an autophagy-specific phosphatidylinositol 3-kinase complex
YBR283C	SSH1, involved in co-translational protein translocation in the ER
YCL010C	SGF29, probable 29kKDa Subunit of SAGA histone acetyltransferase complex
YCR009C	RVS161, roles in endocytosis and in cell fusion
YCR020W-B	HTL1, subunit of the RSC chromatin remodeling complex
YDL033C	SLM3, tRNA-specific 2-thiouridylase
YDL074C	BRE1, E3 ubiquitin ligase for Rad6p
YDL091C	UBX3, UBX domain-containing protein that interacts with Cdc48p
YDL099W	BUG1, involved in ER to Golgi transport
YDL113C	ATG20, involved in localization of membranes to the preautophagosome
YDL135C	RDI1, Rho GDP dissociation inhibitor
YDL146W	LDB17, unknown function
YDL160C	DHH1, cytoplasmic DExD/H-box helicase
YDL192W	ARF1, GTPase of the Ras superfamily involved in coated vesicles formation
YDR006C	SOK1, involved in cAMP-mediated signaling; localized to the nucleus
YDR025W	RPS11A, component of the small (40S) ribosomal subunit

YDR069C	DOA4, ubiquitin-specific protease
YDR078C	SHU2, involved in homologous recombination repair
YDR129C	SAC6, actin filament bundling protein, fimbrin; essential for polarized secretion
YDR173C	ARG82, Inositol polyphosphate multikinase (IPMK)
YDR264C	AKR1, palmitoyl transferase involved in protein palmitoylation
YDR283C	GCN2, protein kinase
YDR295C	HDA2, class II histone deacetylase complex
YDR349C	YPS7, putative GPI-anchored aspartic protease
YDR378C	LSM6, possibly involved in processing tRNA, snoRNA, and rRNA
YDR392W	SPT3, SAGA-like transcriptional regulatory complex
YDR448W	ADA2, component of the histone acetyltransferase complexes
YDR507C	GIN4, serine/threonine-protein kinase required for septin organization at the bud neck
YDR518W	EUG1, protein disulfide isomerase
YDR523C	SPS1, putative protein serine/threonine kinase
YDR533C	HSP 38, possible chaperone and cysteine protease
YEL029C	BUD16, involved in bud-site selection and telomere maintenance
YEL044W	IES6, associates with the INO80 chromatin remodeling complex
YER028C	MIG3, probable transcriptional repressor
YER103W	SSA4, heat shock protein that is highly induced upon stress
YER110C	KAP123, karyopherin beta
YER114C	BOI2, protein implicated in polar growth, functionally redundant with Boi1p
YER122C	GLO3, GTPase-activating protein (GAP) for ADP-ribosylation factors
YER131W	RPS26B, component of the small (40S) ribosomal subunit
YER155C	BEM2, Rho GTPase activating protein; control of cytoskeleton organization
YER169W	RPH1, transcriptional repressor of PHR1
YFR019W	FAB1, involved in orientation or separation of mitotic chromosomes
YGL017W	ATE1, arginyl-tRNA-protein transferase
YGL025C	PGD1, component of RNA polymerase II holoenzyme
YGL038C	OCH1, alpha-1,6-mannosyltransferase
YGL246C	RAI1, nuclear protein required for pre-rRNA processing
YGL251C	HFM1, meiosis specific DNA helicase
YGR262C	BUD32, may be involved in polar bud-site selection
YHR006W	STP2, transcription factor; activates transcription of amino acid permease genes
YIL008W	URM1, ubiquitin-like protein with only weak sequence similarity to ubiquitin
YIL047C	SYG1, member of the divalent anion:Na+ (DASS) family of membrane transporters
YJL052W	TDH1, glyceraldehyde-3-phosphate dehydrogenase, isozyme 1
YJL124C	LSM1, involved in degradation of cytoplasmic mRNAs
YJR004C	SAG1, alpha-agglutinin of alpha-cells
YJR104C	SOD1, cytosolic superoxide dismutase
YKL081W	TEF4, translation elongation factor EF-1 gamma
YKL114C	APN1, major apurinic/apyrimidinic endonuclease, repair of DNA damage
YKL212W	SAC1, lipid phosphoinositide phosphatase of the ER and Golgi
YLL042C	ATG10, E2-like conjugating enzyme, involved in autophagy
YLR025W	SNF7, endosomal sorting complex
YLR070C	XYL2, xylitol dehydrogenase, converts xylitol to D-xylulose
YLR114C	AVL9, involved in exocytic transport from the Golgi
YLR144C	ACF2, beta-1,3-endoglucanase; probable role in cortical actin cytoskeleton assembly
YLR234W	TOP3, DNA topoisomerase III
YLR350W	ORM2, human ortholog is located in the endoplasmic reticulum
YLR394W	CST9, SUMO E3 ligase; required for synaptonemal complex formation
YML088W	UFO1, F-box protein, subunit of the Skp1-Cdc53-F-box (SCF) E3 ubiquitin ligase
YML094W	GIM5, prefoldin subunit 5, component of the Gim protein complex
YML112W	CTK3, C-terminal domain (RNA polymerase II CTD) kinase gamma subunit
YMR070W	MOT3, nuclear transcription factor; e.g. repression of ergosterol biosynthetic genes
YMR071C	TVP18, integral membrane protein localized to late Golgi vesicles
YMR077C	VPS20, myristoylated subunit of the endosomal sorting complex
YMR138W	CIN4, GTP-binding protein involved in chromosome segregation
YMR263W	SAP30, subunit of a histone deacetylase complex
YNL064C	YDJ1, involved in protein import into mitochondria and ER
YNL084C	END3, EH domain-containing protein involved in endocytosis
YNL159C	ASI2, integral inner nuclear membrane protein
YNL160W	YGP1, cell wall-related secretory glycoprotein

YNL225C	CNM67, component of the spindle pole body outer plaque
YNL297C	MON2, role in endocytosis and vacuole integrity
YOL001W	PHO80, cyclin that interacts with Pho85p protein kinase
YOL004W	SIN3, component of the Sin3p-Rpd3p histone deacetylase complex
YOL051W	GAL11, component of RNA polymerase II holoenzyme
YOL100W	PKH2, serine/threonine protein kinase involved in endocytosis
YOL148C	SPT20, component of the nucleosomal histone acetyltransferase
YOR127W	RGA1, GTPase-activating protein for Cdc42p
YOR141C	ARP8, nuclear actin-related protein involved in chromatin remodeling
YOR155C	ISN1, inosine 5'-monophosphate (IMP)-specific 5'-nucleotidase, breakdown of IMP
YOR375C	GDH1, NADP(+)-dependent glutamate dehydrogenase
YOR380W	RDR1, transcriptional repressor, control of multidrug resistance
YPL174C	NIP100, mitotic spindle positioning protein
YPL268W	PLC1, phosphoinositide-specific phospholipase C
YPR066W	UBA3, Rub1-activating enzyme, similar to ubiquitin-activating E1 protein
YPR072W	NOT5, subunit of the CCR4-NOT complex, which is a global transcriptional regulator

UNKNOWN PROTEINS (63 GENES)

Unknown function (41 genes)

YBL044W	Unknown function
YCL001W-A	Unknown function
YCR004C	Unknown function, located in mitochondria
YDL012C	Plasma membrane protein of unknown function
YDL049C	Unknown function
YDL063C	Unknown function
YDL104C	Putative metalloprotease
YDL114W	Putative protein of unknown function with similarity to acyl-carrier-protein reductases
YDL119C	Unknown function, located in mitochondria
YDL129W	Unknown function
YDL133W	Unknown function
YDL157C	Unknown function; detected in highly purified mitochondria in high-throughput studies
YDL167C	Unknown function
YDR042C	Unknown function
YDR316W	Protein integral to the mitochondrial membrane
YDR458C	Unknown function; GFP-fusion protein in nuclear periphery
YDR512C	Unknown function
YER077C	Unknown function
YGL220W	Unknown function
YGR243W	Unknown function, localized to mitochondria
YHR009C	Unknown function
YHR039C	Unknown function, GFP-fusion protein in endoplasmic reticulum
YHR059W	Unknown function
YIL015C-A	Unknown function
YIL060W	Unknown function, 100% identical with YHR145C
YJL023C	PET130, protein is detected in highly purified mitochondria in high-throughput studies
YJL184W	Unknown function
YJR120W	Unknown function
YLR125W	Unknown function
YLR149C	Unknown function
YLR218C	Unknown function
YML072C	Unknown function, contains three calcium and lipid binding domains
YML087C	Unknown function
YMR063W	Unknown function
YMR067C	UBX4, UBX domain-containing protein, unknown function
YMR084W	Unknown function
YMR184W	Unknown function
YMR244C-A	Unknown function
YNL080C	Unknown function
YOL083W	Unknown function
YOL138C	Unknown function

Questionable ORFs (22 genes)

YBL012C	Dubious ORF, overlaps with SCT1
YBL053W	Dubious ORF, overlaps with SAS3
YBL062W	Dubious ORF, overlaps with SKT5
YDL032W	Dubious ORF, overlaps with YDL033C and DBP10 promoter
YDL062W	Dubious ORF, overlaps uncharacterized ORF YDL063C
YDR010C	Dubious ORF
YDR269C	Dubious ORF, overlaps with CCC2
YDR271C	Dubious ORF, overlaps with CCC2
YDR491C	Dubious ORF, overlaps with IZH1
YGL165C	Dubious ORF, overlaps with CUP2
YHL005C	Dubious ORF, overlaps with MRP4 promoter
YHR049C-A	Dubious ORF
YJL022W	Dubious ORF, overlaps with PET130
YJL175W	Dubious ORF, overlaps with SWI3
YJR087W	Dubious ORF, overlaps the gene STE18 and uncharacterized ORF YJR088C
YLR294C	Dubious ORF, overlaps with ATP14
YMR245W	Dubious ORF
YNR025C	Dubious ORF, overlaps with YNR024w
YOR199W	Dubious ORF, overlaps with MRM1
YOR318C	Dubious ORF unlikely to encode a protein
YOR333C	Dubious ORF, overlaps with MRS2
YPL136W	Dubious ORF, overlaps with GIP3

19 additional *PET* genes (deletion mutants were not present in all three libraries)

6 *PET* genes found in two of two screens

YDR388W	RVS167, actin-associated protein, homolog of mammalian amphiphysin
YDR405W	MRP20, mitochondrial ribosomal protein
YFL036W	RPO41, RNA polymerase, mitochondrial
YKL194C	MST1, mitochondrial threonyl tRNA synthase
YNL213C	RRG9, unknown function, located in mitochondria
YOL143C	RIB4, riboflavin biosynthesis pathway enzyme

6 *PET* genes found in one of one screens

YBR039W	ATP3, gamma subunit of the F1 sector of mitochondrial F1F0 ATP synthase
YBR122C	MRPL36, mitochondrial ribosomal protein of the large subunit
YJL062W-A	RRG10, unknown function, GFP-fusion protein localizes to the mitochondria
YMR231W	PEP5, vacuolar protein required for vacuole biogenesis
YNL185C	MRPL19, mitochondrial ribosomal protein
YPL189C-A	COA2, cytochrome c oxidase assembly factor

7 *PET* genes found in one of two screens

YBR097W	VPS15, serine/threonine protein kinase involved in vacuolar protein sorting
YDL039C	PRM7, pheromone-regulated protein
YDL057W	Unknown function
YDL181W	INH1, inhibits ATP hydrolysis by the F1F0-ATP synthase
YEL059C-A	SOM1, subunit of the mitochondrial inner membrane peptidase, maturation of proteins
YER014W	HEM14, protoporphyrinogen oxidase
YGL024W	Dubious ORF, overlaps with PGD1
