# Supplementary material for: Genome-wide deletion mutant analysis reveals genes required for respiratory growth, mitochondrial genome maintenance and mitochondrial protein synthesis in Saccharomyces cerevisiae
Source: Genome Biol. 2009 Sep 14;10(9):R95. doi: 10.1186/gb-2009-10-9-r95 (PMC2768984; doi:10.1186/gb-2009-10-9-r95)
Supplement: Additional data file 4 — pet genes producing growth defects only on specific carbon sources. [file gb-2009-10-9-r95-S4.PDF]

Supplemental table 4. *pet* genes producing growth defects only on specific carbon sources. The list indicates systematic and standard names and a brief description of gene function according to the Saccharomyces Genome Database and manual annotation.

Growth defect on glycerol, but not on ethanol or lactate containing media

YBR026C	ETR1, localized to in mitochondria, where it has a probable role in fatty acid synthesis
YDR269C	Dubious ORF, overlaps with CCC2
YDR271C	Dubious ORF, overlaps with CCC2
YGL244W	RTF1, pol II transcription elongation factor, regulates DNA binding properties of TBP
YGL251C	HFM1, meiosis specific DNA helicase
YJR040W	GEF1, voltage-gated chloride channel
YPL188W	POS5, mitochondrial NADH kinase; required for the response to oxidative stress

Growth defect on glycerol or ethanol, but not on lactate containing media

YBR283C	SSH1, involved in co-translational protein translocation in the ER
YDL039C	PRM7, pheromone-regulated protein
YDR025W	RPS11A, component of the small (40S) ribosomal subunit
YHR049C-A	Dubious ORF
YJR113C	RSM7, mitochondrial ribosomal protein
YNL005C	MRP7, mitochondrial ribosomal protein
YNL170W	Dubious ORF, overlaps with PSD1

Growth defect on glycerol or lactate, but not on ethanol containing media

YPR134W	MSS18, involved in splicing a15beta intron of the mitochondrial COX1 transcript
