# Supplementary material for: Genome-wide deletion mutant analysis reveals genes required for respiratory growth, mitochondrial genome maintenance and mitochondrial protein synthesis in Saccharomyces cerevisiae
Source: Genome Biol. 2009 Sep 14;10(9):R95. doi: 10.1186/gb-2009-10-9-r95 (PMC2768984; doi:10.1186/gb-2009-10-9-r95)
Supplement: Additional data file 5 — Mutants belonging to four classes of pet genes. [file gb-2009-10-9-r95-S5.PDF]

**Supplemental table 5.** Classes of *pet* genes. The list indicates systematic and standard names of genes.

**Class I *pet* mutants**

YAL039C/CYC3
YBL019W/APN2
YBL021C/HAP3
YBL080C/PET112
YBL090W/MRP21
YBR039W/ATP3
YBR146W/MRPS9
YBR179C/FZO1
YBR251W/MRPS5
YBR268W/MRPL37
YBR282W/MRPL27
YCR003W/MRPL32
YCR024C/SLM5
YCR071C/IMG2
YDL044C/MTF2
YDL045W-A/MRP10
YDL129W
YDL133W
YDR065W/RRG1
YDR079W/PET100
YDR114C
YDR115W
YDR175C/RSM24
YDR194C/MSS116
YDR296W/MHR1
YDR337W/MRPS28
YDR347W/MRP1
YDR350C/ATP22
YDR377W/ATP17
YEL050C/RML2
YER050C/RSM18
YER070W/RNR1
YER154W/OXA1
YFL016C/MDJ1
YGL129C/RSM23
YGL143C/MRF1
YGL240W/DOC1
YGR076C/MRPL25
YGR102C
YGR150C/RRG2
YGR167W/CLC1
YGR171C/MSM1
YGR215W/RSM27
YHL038C/CBP2
YHR011W/DIA4
YHR038W/RRF1
YHR091C/MSR1
YHR120W/MSH1
YHR147C/MRPL6
YHR168W/MTG2
YJL063C/MRPL8
YJL096W/MRPL49
YJL102W/MEF2
YJR144W/MGM101
YKL003C/MRP17
YKL114C/APN1
YKL134C/OCT1
YKL138C/MRPL31
YKL155C/RSM22
YKL169C
YKL170W/MRPL38
YKL194C/MST1
YKR006C/MRPL13
YLL027W/ISA1
YLL033W/RRG4 (IRC19)
YLR067C/PET309
YLR069C/MEF1
YLR070C/XYL2
YLR091W/RRG5
YLR139C/SLS1
YLR295C/ATP14
YLR304C/ACO1
YLR312W-A/MRPL15
YLR369W/SSQ1
YLR382C/NAM2
YLR439W/MRPL4
YML061C/PIF1
YMR015C/ERG5
YMR064W/AEP1
YMR089C/YTA12
YMR098C
YMR158W/MRPS8
YMR188C/MRPS17
YMR193W/MRPL24
YMR228W/MTF1
YMR267W/PPA2
YMR282C/AEP2
YMR287C/DSS1
YMR293C/RRG6 (HER2)
YNL073W/MSK1
YNL081C/SWS2
YNL177C/MRPL22
YNL184C
YNL185C/MRPL19
YNL213C/RRG9
YNL252C/MRPL17
YNR037C/RSM19
YOL009C/MDM12
YOL033W/MSE1
YOL083W
YOL095C/HMI1
YOR150W/MRPL23
YOR158W/PET123
YOR187W/TUF1
YOR211C/MGM1
YOR241W/MET7
YOR330C/MIP1
YOR375C/GDH1
YPL013C/MRPS16
YPL078C/ATP4
YPL097W/MSY1
YPL104W/MSD1
YPL148C/PPT2
YPL173W/MRPL40
YPL254W/HFI1
YPL271W/ATP15
YPR067W/ISA2
YPR116W/RRG8

**Class II *pet* mutants**

YDL033C/SLM3
YDL099W/BUG1
YDL107W/MSS2
YDL114W
YDR270W/CCC2
YDR364C/CDC40
YDR458C/HEH2
YEL059C-A/SOM1
YGL070C/RPB9
YJL003W/COX16
YKL040C/NFU1
YKL080W/VMA5
YKL109W/HAP4
YLL018C-A/COX19
YLR294C
YLR337C/VRP1
YML110C/COQ5
YOL008W/COQ10
YOL051W/GAL11
YOR200W
YOR221C/MCT1
YOR305W/RRG7
YPL172C/COX10

**Class III *pet* mutants**

YAL009W/SPO7
YAL010C/MDM10
YAL013W/DEP1
YAL044C/GCV3
YBL045C/COR1
YBL082C/ALG3
YBL093C/ROX3
YBL099W/ATP1
YBL100C
YBR003W/COQ1
YBR026C/ETR1
YBR097W/VPS15
YBR127C/VMA2
YBR283C/SSH1
YBR289W/SNF5
YCL001W-A
YCL007C
YCR020W-B/HTL1
YCR046C/IMG1
YDL039C/PRM7
YDL067C/COX9
YDL068W
YDL077C/VMA6
YDL185W/VMA1
YDR010C
YDR025W/RPS11A
YDR116C/MRPL1
YDR148C/KGD2
YDR197W/CBS2
YDR204W/COQ4
YDR230W
YDR237W/MRPL7
YDR269C
YDR271C
YDR298C/ATP5
YDR349C/YPS7
YDR375C/BCS1
YDR448W/ADA2
YDR529C/QCR7
YEL024W/RIP1
YEL027W/CUP5
YEL051W/VMA8
YER014C-A/BUD25
YER017C/AFG3
YER061C/CEM1
YER145C/FTR1
YGL071W/RCS1
YGL237C/HAP2
YGL244W/RTF1
YGL251C/HFM1
YGR020C/VMA7
YGR062C/COX18
YGR105W/VMA21
YGR112W/SHY1
YGR155W/CYS4
YGR220C/MRPL9
YGR222W/PET54
YGR262C/BUD32
YHR026W/PPA1
YHR039C/MSC7
YHR039C-B/VMA10
YHR049C-A
YHR051W/COX6
YHR060W/VMA22
YHR067W/HTD2
YIL125W/KGD1
YIL157C/COA1
YIR021W/MRS1
YJL046W/RRG3
YJL062W-A/RRG10
YJL120W
YJL121C/RPE1
YJL124C/LSM1
YJL176C/SWI3
YJL180C/ATP12
YJL184W/GON7
YJL209W/CBP1
YJR040W/GEF1
YJR077C/MIR1
YJR113C/RSM7
YJR121W/ATP2
YJR122W/CAF17
YKL016C/ATP7
YKL055C/OAR1
YKL119C/VPH2
YKR085C/MRPL20
YLL041C/SDH2
YLR038C/COX12
YLR201C/COQ9
YLR202C
YLR203C/MSS51
YLR447C/VMA6
YML120C/NDI1
YMR021C/MAC1
YMR035W/IMP2
YMR097C/MTG1
YMR150C/IMP1
YMR151W/YIM2
YMR256C/COX7
YMR257C/PET111
YMR286W/MRPL33
YNL005C/MRP7
YNL052W/COX5A
YNL138W/SRV2
YNL170W
YNL315C/ATP11
YNR020C/ATP23
YNR041C/COQ2
YNR042W
YNR045W/PET494
YOL071W/EMI5
YOL096C/COQ3
YOR036W/PEP12
YOR065W/CYT1
YOR331C
YOR332W/VMA4
YOR350C/MNE1
YOR358W/HAP5
YOR380W/RDR1
YPL031C/PHO85
YPL045W/VPS16
YPL059W/GRX5
YPL132W/COX11
YPL136W
YPL189C-A
YPL188W/POS5
YPL215W/CBP3
YPL234C/VMA11
YPL262W/FUM1
YPR036W/VMA13
YPR066W/UBA3
YPR099C
YPR123C
YPR191W/QCR2

**Class IV *pet* mutants**

YAL026C/DRS2
YBL031W/SHE1
YBL032W/HEK2
YBL036C
YBL046W/PSY4
YBL053W
YBL057C/PTH2
YBL062W
YBR128C/ATG14
YCL010C/SGF29
YCR028C-A/RIM1
YDL012C
YDL056W/MBP1
YDL091C/UBX3
YDL157C
YDL192W/ARF1
YDR491C
YDR523C/SPS1
YER087W
YER114C/BOI2
YER131W/RPS26B
YER155C/BEM2
YGL017W/ATE1
YGL135W/RPL1B
YGL165C
YGL206C/CHC1
YGL218W
YGR180C/RNR4
YGR243W/FMP43
YHR006W/STP2
YHR009C
YLL042C/ATG10
YLR125W
YLR144C/ACF2
YLR260W/LCB5
YLR270W/DCS1
YML087C
YMR070W/MOT3
YMR072W/ABF2
YMR077C/VPS20
YNL159C/ASI2
YOR127W/RGA1
YOR155C/ISN1
YOR318
