# Supplementary material for: Genome-wide deletion mutant analysis reveals genes required for respiratory growth, mitochondrial genome maintenance and mitochondrial protein synthesis in Saccharomyces cerevisiae
Source: Genome Biol. 2009 Sep 14;10(9):R95. doi: 10.1186/gb-2009-10-9-r95 (PMC2768984; doi:10.1186/gb-2009-10-9-r95)
Supplement: Additional data file 6 — Quantification of loss of mtDNA in class IV pet mutants. [file gb-2009-10-9-r95-S6.PDF]

Supplemental table 6. Loss of mtDNA in class IV *pet* mutants. mtDNA was replenished in class IV mutants by cytoduction and visualized by DAPI staining. Then mutants were kept replicatively growing in YPD medium for 10 days to allow for loss of mtDNA, and the mtDNA content was determined by DAPI staining. At least 100 cells were analyzed per strain. Five strains did not yield cytoductants and were not analyzed further (n.d.).

**deleted gene (systematic/standard name)**	**mtDNA content immediately after cytoduction (% of cells)**		**mtDNA content after 10 days of growth in glucose-containing medium (% of cells)**		
	**normal**	**[*rho*^{0}]**	**normal**	**reduced**	**[*rho*^{0}]**
Wild type	99,2	0,8	92,2	2,9	4,9
YAL026c/DRS2	92,2	7,8	82,2	0,0	17,8
YBL031w/SHE1	100,0	0,0	97,3	0,0	2,7
YBL032w/HEK2	100,0	0,0	83,1	12,3	4,6
YBL036c	96,2	3,8	68,3	0,0	31,7
YBL046w/PSY4	96,2	3,8	94,8	0,0	5,2
YBL053w	99,3	0,7	65,2	23,9	10,9
YBL057c/ PTH2	96,5	3,5	34,1	18,0	47,9
YBL062w	96,1	3,9	78,4	21,6	0,0
YBR128c/ATG14	100,0	0,0	98,3	1,7	0,0
YCL010c/SGF29	100,0	0,0	51,4	33,3	15,3
YCR028c-A/RIM1	n.d.			n.d.	
YDL012c	97,4	2,6	68,4	10,8	20,8
YDL056w/MBP1	100,0	0,0	55,6	28,2	16,2
YDL091c/UBX3	99,2	0,8	34,2	32,2	33,6
YDL157c	99,0	1,0	28,2	32,2	39,6
YDL192w/ARF1	97,1	2,9	69,2	18,0	12,8
YDR491c	97,5	2,5	88,0	0,0	12,0
YDR523c/SPS1	100,0	0,0	21,4	29,1	49,5
YER087w	n.d.			n.d.	
YER114c/BOI2	98,0	2,0	27,9	15,0	57,1
YER131w/RPS26B	98,7	1,3	54,4	22,8	22,8
YER155c/BEM2	99,0	1,0	81,5	12,0	6,5
YGL017w/ATE1	91,8	8,2	85,3	8,8	5,9
YGL135w/RPL1B	95,3	4,7	83,0	3,6	13,4
YGL165c	100,0	0,0	74,0	10,2	15,8
YGL206c/CHC1	n.d.			n.d.	
YGL218w	n.d.			n.d.	
YGR180c/RNR4	n.d.			n.d.	
YGR243w/FMP43	100,0	0,0	100,0	0,0	0,0
YHR006w/STP2	100,0	0,0	14,0	31,2	54,8
YHR009c	99,0	1,0	36,0	24,7	39,3
YLL042c/ATG10	100,0	0,0	26,4	10,0	63,6
YLR125w	100,0	0,0	84,3	6,3	9,4
YLR144c/ACF2	100,0	0,0	83,0	13,0	4,0
YLR260w/LCB5	100,0	0,0	82,0	14,0	4,0
YLR270w/DCS1	100,0	0,0	68,3	13,7	18,0
YML087c	100,0	0,0	85,9	0,0	14,1
YMR070w/MOT3	100,0	0,0	63,8	19,8	16,4
YMR072w/ABF2	92,6	7,4	0,0	0,0	100,0

YMR077c/VPS20	100,0	0,0	48,3	27,1	24,6
YNL159c/ASI2	100,0	0,0	75,2	18,4	6,4
YOR127w/RGA1	100,0	0,0	67,5	18,0	14,5
YOR155c/ISN1	100,0	0,0	53,2	32,5	14,3
YOR318c	100,0	0,0	66,2	24,1	9,7
