# Supplementary material for: Genome-wide deletion mutant analysis reveals genes required for respiratory growth, mitochondrial genome maintenance and mitochondrial protein synthesis in Saccharomyces cerevisiae
Source: Genome Biol. 2009 Sep 14;10(9):R95. doi: 10.1186/gb-2009-10-9-r95 (PMC2768984; doi:10.1186/gb-2009-10-9-r95)
Supplement: Additional data file 7 — pet genes dispensable for respiration. [file gb-2009-10-9-r95-S7.PDF]

**Supplemental table 7.** Genes dispensable for respiration. The list indicates systematic and standard names of genes deleted in class III mutants that are able to grow on non-fermentable carbon sources after relief of catabolite repression.

YAL009W/SPO7
YAL010C/MDM10
YAL013W/DEP1
YAL044C/GCV3
YBL082C/ALG3
YBL093C/ROX3
YBR026C/ETR1
YBR097W/VPS15
YBR127C/VMA2
YBR283C/SSH1
YBR289W/SNF5
YCL001W-A
YCL007C
YCR020W-B/HTL1
YCR046C/IMG1
YDL039C/PRM7
YDL067C/COX9
YDL068W
YDL077C/VMA6
YDL185W/VMA1
YDR010C
YDR025W/RPS11A
YDR116C/MRPL1
YDR148C/KGD2
YDR230W
YDR237W/MRPL7
YDR269C
YDR271C
YDR298C/ATP5
YDR349C/YPS7
YDR448W/ADA2
YER014C-A/BUD25
YER017C/AFG3
YGL237C/HAP2
YGL244W/RTF1
YGL251C/HFM1
YGR155W/CYS4
YGR262C/BUD32
YHR039C/MSC7
YHR049C-A
YHR060W/VMA22
YHR067W/HTD2
YIL125W/KGD1
YIL157C/COA1
YJL046W/RRG3
YJL120W
YJL121C/RPE1
YJL124C/LSM1
YJL176C/SWI3
YJL184W/GON7
YJR040W/GEF1
YJR077C/MIR1
YJR113C/RSM7
YKL055C/OAR1
YLR038C/COX12
YML120C/NDI1
YNL005C/MRP7
YNL052W/COX5A
YNL138W/SRV2
YNL170W
YNL315C/ATP11
YNR020C/ATP23
YOR036W/PEP12
YOR331C
YOR350C/MNE1
YOR380W/RDR1
YPL031C/PHO85
YPL045W/VPS16
YPL059W/GRX5
YPL136W
YPL188W/POS5
YPL215W/CBP3
YPL234C/VMA11
YPL262W/FUM1
YPR066W/UBA3
YPR099C
YPR191W/QCR2
