# Supplementary material for: Genome-wide deletion mutant analysis reveals genes required for respiratory growth, mitochondrial genome maintenance and mitochondrial protein synthesis in Saccharomyces cerevisiae
Source: Genome Biol. 2009 Sep 14;10(9):R95. doi: 10.1186/gb-2009-10-9-r95 (PMC2768984; doi:10.1186/gb-2009-10-9-r95)
Supplement: Additional data file 8 — Genes required for respiratory activity in class I and III pet mutants that show a wild-type pattern of mitochondrial translation products. [file gb-2009-10-9-r95-S8.PDF]

Supplemetal table 8. Genes required for respiratory activity in class I and III *pet* mutants that show a wild type pattern of mitochondrial translation products. The cellular roles of the proteins are indicated according to the Saccharomyces Genome Database (SGD) or manually annotated.

**Genes encoding mitochondrial proteins**		
YBL045C	*COR1*	Ubiquinol cytochrome *c* reductase core protein 1
YBL080C	*PET112*	Protein required for mitochondrial translation
YBL099W	*ATP1*	Alpha subunit of F_1-ATP synthase
YBR003W	*COQ1*	Catalyzes the first step in ubiquinone (coenzyme Q) biosynthesis
YBR039W	*ATP3*	Gamma subunit of the F_1 sector of mitochondrial F_1F_0 ATP synthase
YDR204W	*COQ4*	Involved in biosynthesis of coenzyme Q
YDR350C	*ATP22*	Required for assembly of the F_0 sector of mitochondrial ATP synthase
YDR375C	*BCS1*	Required for expression of functional Rieske iron-sulfur protein
YDR377W	*ATP17*	ATP synthase subunit f
YDR529c	*QCR7*	Subunit 7 of the ubiquinol cytochrome *c* reductase complex
YEL024W	*RIP1*	Ubiquinol cytochrome *c* reductase iron-sulfur protein
YER061C	*CEM1*	Beta-ketoacyl-ACP synthase, mitochondrial
YFL016C	*MDJ1*	DnaJ co-chaperone involved in mitochondrial biogenesis
YGR062C	*COX18*	Required for activity of mitochondrial cytochrome *c* oxidase
YGR112W	*SHY1*	Required for assembly of cytochrome *c* oxidase complex
YJL180C	*ATP12*	F_1-ATP synthase assembly protein
YJR121W	*ATP2*	Beta subunit of F_1-ATP synthase
YLL027W	*ISA1*	Mitochondrial protein required for normal iron metabolism
YLL041C	*SDH2*	Iron-sulfur protein subunit of succinat dehydrogenase
YLR201C	*COQ9*	Required for ubiquinone biosynthesis
YLR369W	*SSQ1*	Mitochondrial Hsp70 involved in biogenesis of iron-sulfur proteins
YLR382C	*NAM2*	Leucyl-tRNA synthetase, mitochondrial
YMR035W	*IMP2*	Catalytic subunit of the mitochondrial inner membrane protease Imp
YMR150C	*IMP1*	Catalytic subunit of the mitochondrial inner membrane protease Imp
YMR256C	*COX7*	Cytochrome *c* oxidase, subunit VII
YMR257C	*PET111*	Required for mitochondrial translation of *COX2* mRNA
YNR041C	*COQ2*	Para-hydroxybenzoate-polyprenyltransferase
YOL009C	*MDM12*	Mitochondrial morphology and inheritance protein
YPL013C	*MRPS16*	Mitochondrial ribosomal protein
YPL132W	*COX11*	Required for delivery of copper to Cox1
YPL189C-A	*COA2*	Cytochrome *c* oxidase assembly factor
YPR067W	*ISA2*	Mitochondrial protein required for iron metabolism
**Genes required for vacuolar functions**		
YEL027W	*CUP5*	V-ATPase 16 kDa proteolipid subunit of membrane (V_0) sector
YGR020C	*VMA7*	V-ATPase 14 kDa subunit of the catalytic (V_0) sector
YGR105W	*VMA21*	Required for vacuolar V-ATPase assembly
YHR026W	*PPA1*	V-ATPase proteolipid
YHR039C-B	*VMA10*	V-ATPase 13 kDa subunit
YKL119C	*VPH2*	V-ATPase assembly protein
YLR447C	*VMA6*	V-ATPase 36 kDa subunit
YOR332W	*VMA4*	V-ATPase hydrophilic subunit (subunit E)
YPR036W	*VMA13*	V-ATPase 54 kDa subunit of V_1 sector
**Other genes encoding known proteins**		
YBL021C	*HAP3*	Transcriptional activator of respiratory gene expression
YER070W	*RNR1*	Ribonucleosid-diphosphate-reductase, large (R1) subunit
YGR167W	*CLC1*	Clathrin light chain
YJR122W	*CAF17*	Component of the CCR4 transcription complex
YMR015C	*ERG5*	C-22 sterol desaturase

YMR021C	*MAC1*	Copper-sensing transcription factor
YOR241W	*MET7*	Required for methionine synthesis and for maintenance of mtDNA
YOR358W	*HAP5*	Transcriptional activator of respiratory gene expression
YOR375C	*GDH1*	$NADP^+$-dependent glutamate dehydrogenase
**ORFs encoding unknown proteins**		
YBL100C		Dubious open reading frame, overlaps the 5' end of *ATP1*
YDL129W		Unknown function
YDL133W		Unknown function
YLL033W	*RRG4*	*IRC19*, Unknown function
YLR202C		Dubious ORF, overlaps with *COQ9*
YMR151W		Dubious ORF, overlaps with *IMP1*
YNL213C	*RRG9*	Unknown function; protein is detected in highly purified mitochondria
YNR042W		Dubious ORF, overlaps with *COQ2*
YOL071W		Unknown function
YOL083W		Unknown function
YPR123C		Dubious ORF, overlaps with *CTR1*
