# Supplementary material for: Genome-wide deletion mutant analysis reveals genes required for respiratory growth, mitochondrial genome maintenance and mitochondrial protein synthesis in Saccharomyces cerevisiae
Source: Genome Biol. 2009 Sep 14;10(9):R95. doi: 10.1186/gb-2009-10-9-r95 (PMC2768984; doi:10.1186/gb-2009-10-9-r95)
Supplement: Additional data file 9 — Genes possibly affecting mitochondrial function in combination with acquired defects. [file gb-2009-10-9-r95-S9.PDF]

Supplemental table 9. Genes possibly affecting mitochondrial function in combination with acquired defects. The list indicates systematic and standard names of genes deleted in class II *pet* mutants that are both rescued by mating with Δ*mip1* as well as cytoduction. Mutants highlighted with an asterisk showed restoration of respiratory growth also after cytoduction with a [*rho*^{0}] donor strain. The cellular roles of the proteins are indicated according to the Saccharomyces Genome Database (SGD) or manually annotated.

**Genes encoding components involved cytochrome c oxidase assembly**		
YDL107W	*MSS2*	Involved in membrane insertion of C-terminus of Cox2
YJL003W	*COX16*	Required for assembly of cytochrome *c* oxidase
*YLL018C-A	*COX19*	Required for assembly of cytochrome *c* oxidase
YPL172C	*COX10*	Required for activity of cytochrome *c* oxidase
**Genes encoding other mitochondrial proteins**		
*YDL033C	*SLM3*	Responsible for 2-thiolation of the wobble base of mitochondrial tRNAs
YEL059C-A	*SOM1*	Subunit of the mitochondrial inner membrane peptidase
*YKL040C	*NFU1*	Protein involved in iron metabolism in mitochondria
YML110C	*COQ5*	Involved in ubiquinone (Coenzyme Q) biosynthesis
*YOL008W	*COQ10*	Coenzyme Q (ubiquinone) binding protein
YOR221C	*MCT1*	Putative component of a type-II mitochondrial fatty acid synthase
**Genes encoding extramitochondrial proteins**		
YDL099W	*BUG1*	Cis-golgi localized protein involved in ER to Golgi transport
*YDR270W	*CCC2*	Copper transporting P-type ATPase
*YDR364C	*CDC40*	Pre-mRNA splicing factor
*YDR458C	*HEH2*	Inner nuclear membrane (INM) protein
*YGL070C	*RPB9*	RNA polymerase II subunit B12.6
*YKL080W	*VMA5*	Subunit of the vacuolar V-ATPase
YKL109W	*HAP4*	Global regulator of respiratory gene expression
*YLR337C	*VRP1*	Involved in cytoskeletal organization and cytokinesis
*YOL051W	*GAL11*	Subunit of the RNA polymerase II mediator complex
**Uncharacterized genes**		
YDL114W		Unknown function
YLR294C		Dubious ORF, partially overlaps with *ATP14*
YOR200W		Dubious ORF, partially overlaps with *MRM1*
YOR305W	*RRG7*	Unknown function
