# Supplementary material for: Genome-wide deletion mutant analysis reveals genes required for respiratory growth, mitochondrial genome maintenance and mitochondrial protein synthesis in Saccharomyces cerevisiae
Source: Genome Biol. 2009 Sep 14;10(9):R95. doi: 10.1186/gb-2009-10-9-r95 (PMC2768984; doi:10.1186/gb-2009-10-9-r95)
Supplement: Additional data file 10 — Primers used to confirm the identity of yeast deletion mutants. [file gb-2009-10-9-r95-S10.PDF]

Supplemental table S10. Primers used to confirm the identity of yeast deletion mutants.

**Forward primers**	
Homologous to kanMX4 deletion cassette	
Kan-cassette	5' CCG GAT TCA GTC ACT CAT GG
Homologous to coding regions	
WT-COX10	5' GAC GTG ATT CGG GCG TGA TTA ATA TTC C
WT-COX16	5' CGG GTT ATG GCC GTA CAC AAG TTA TTA G
WT-COX19	5' GGA GTT GAG AAA CGA GAA AAT CCA AAT AAA GC
WT-MSS2	5' GGA ATT CGT TTT TAG ATT TTA ACA ACA ACA ATA TGA GGG
WT-YAL012W	5' GGC CAC TAA TAA CAA GCC ATT GTA CGA G
WT-YAL047C	5' CAG TTA AAT ACT TTG GAC AAC CAA AAG TTA ATA CTA TC
WT-YBL038W	5' GTA GAG TGC CAG TTC GTA CA
WT-YBR163W	5' AGG CTC TAA TAC ATT TGA TAT GAC CGC GTT C
WT-YDL202W	5' GTC CAT TAC AAT AAT CTT TCC AAA A
WT-YDR231C	5' GGG GTC AAA AGA TCC TCT TAG AAG ACA C
WT-YDR268W	5' CAG CAT TTG GAA CTA ACA AGA CA
WT-YDR323C	5' CTT CCG CTG CAT ACA TAA ACG AAA AAA TCC TAC
WT-YDR332W	5' GTG GGG CCT CCC TTT AAA AGG TAA G
WT-YIL036W	5' CAA TCC CTG GTA CTA CGG CAT GGA AG
WT-YJR090C	5' CGG GCA ACA TAG ATT ACC AAA AAG GGC
WT-YKL148C	5' CAG CCT GGG TTG CCA CAC AAA
WT-YML081C-A	5' GTT GAA AAG ATT CCC TAC CCC TAT CC
WT-YML129C	5' CCA AAT ACG CTT GGT ATA CCA GAG TTA CAG
WT-YMR066W	5' GTC GTC CAT TGT AAA GAA ATT AAT AAA AAG GCA G
WT-YOR205C	5' GGT ATT GAT GTA TTC AAC TCG TGC AAT TCA TC
WT-YPL029W	5' GCA GAC TAT TTC AGA TGA GCT A
WT-YPR047W	5' CTC GAA CTC TGC GGA TGC
WT-YPR124W	5' CTC TTT CAT GAC ATT ATA AGG GCG TTC TTA G
**Reverse primers**	
Homologous to non-coding regions outside the deleted ORF	
cox10	5' GAA AGA TAT AGC TAA GCT AGT AGC ACC TG
cox16	5' GTT GAA TTA TCG GTA TTT CTT CCG GAA GGG
cox19	5' CCG GTA GAT CTG GGA AGT AAA TAC TAA AC
mss2	5' CAA GGA TGA TAC GCT CAA TTT ACT GGA TAC
YAL012W	5' GAT TGC GGA TGA GTA AGC GAA GAG TTA TAG
YAL013W	5' GAA CCA GAG GAG AAA GCC AAC CC
YAL039C	5' GGT TGC GAC ACT TCC CCA GAA GG
YAL047C	5' GGA TCT GAA ATG AGG CAA CCA AAA GAG AG
YBL038W	5' GTT TTT TCT TTG CAG CAT CGA AAT
YBR128C	5' CAC TTG CAC AAT GGC TCT ATC TCC TC
YBR146W	5' GAT ATG GAT CCA GCC ATC CCA CTG
YBR163W	5' CCA AAG AAC TGA ATC TTG TAG AAT TGA AGG AC
YDL077C	5' CCC AGC AAT TCT TCC TTT TTC CCT TAG TC
YDL157C	5' CCA AGA TTA CCC AAA GAC CGT ATC TAT TCC
YDL202W	5' TGA CGA TAA TAT CTC TTC TTT GG
YDR065W	5' GGT ACC CGC TAT GCT ATA AGA GTG CG
YDR231C	5' GAG AGA CTG CTG GAG AAG CA
YDR268W	5' CTC AAC TCC TTG GCC GCA A
YDR323C	5' GAA GCT TCT ATT TAT GTT GAA GCT TCC TTC AG
YDR332W	5' GCA ATC ACC TTT CTT TAT TGC GGG AAT CC
YDR448W	5' CGT AAG GAT CTA CCA GAA TTG TAT TTG AAA ACA G
YDR529C	5' CTA CGA GGA GAA CCC CTC CAC G
YEL051W	5' AAT TTT AGT AGA AAA GAA GCA ACA TAG GGA CC
YGL017W	5' CTT GAC ATT CGC AGA GTA CTT GTC ATC AG
YGR150C	5' GGG CAG TTA AAT TAA GGT CAC CTT GGC
YGR243W	5' CTT ATC CAA TAA GAA CGT AGA TGC
YIL036W	5' GGC ATA GAT TCA TCA ATG ATG GTA ATA AAC ATT TAA GC

YJL046W	5' GGT CCA AAT GGC GAC ATT GAA GAC AC
YJL062W-A	5' CCA TTG GTG CAC GTT GTT GAA GTG G
YJR090C	5' CTA GCT AAT GAC AAA GAC GGT GAT CTG G
YKL148C	5' AAA ATC CTG ACC ATC ACG AAT AC
YLL033W	5' CTA GGG TCT GCC TCC AGC AAG AG
YLR091W	5' CGC CCT TTG AGC TGT TCA CTG CG
YML081C-A	5' CAA GAA AAT CTA CTA CCC TAA TCT
YML129C	5' ATA AAT AAT GTA CAA TTT TAC GGG TG
YMR015C	5' CAT CCT TCT TGG ATT GCA AAA AGA TTA GCT G
YMR066W	5' CCC GAT AGA AGC CAT ATT CAA ATA GCA TTC
YMR098C	5' TTG CCG GGC ATA AGA TCC TTT CTA GTG
YMR293C	5' GGT GTG GCC TTC ACT TTC GGC G
YOL096C	5' GAT GTG GTT CAC GAC CCG TCA CTT G
YOR205C	5' GCA ATT AAG ATT TTC ATC TTT TAG TGA AAA GAT GAA GG
YOR305W	5' CGA TAA GTT CGG TAG GTT TAA CGT CGC
YPL029W	5' TTT CGT CAA AAT CCT TGG ATG C
YPL188W	5' GCT GCA CCA TTT ATC TCA TAA TTA TTG CCT AC
YPR047W	5' TGG TTG AGC AAA TTC GAC GG
YPR116W	5' GAG ACC ACG GTA ACA TAG ACA TTG TAG AT
YPR124W	5' GAT TTC TTT ATG AAA TTT TCT TTA CTC GAA CCT AAA TAT CAC
